# T-cell calcium dynamics visualized in a ratiometric tdTomato-GCaMP6f transgenic reporter mouse

**DOI:** 10.7554/eLife.32417

**Published:** 2017-12-14

**Authors:** Tobias X Dong, Shivashankar Othy, Amit Jairaman, Jonathan Skupsky, Angel Zavala, Ian Parker, Joseph L Dynes, Michael D Cahalan

**Affiliations:** 1 Department of Physiology and BiophysicsUniversity of CaliforniaIrvineUnited States; 2 Department of MedicineUniversity of CaliforniaIrvineUnited States; 3 Department of Neurobiology & BehaviorUniversity of CaliforniaIrvineUnited States; 4 Institute for ImmunologyUniversity of CaliforniaIrvineUnited States; University of OxfordUnited Kingdom

**Keywords:** genetically encoded Ca2+ indicator, Ca2+ signaling, T cell motility, Orai1, two-photon microscopy, Human, Mouse

## Abstract

Calcium is an essential cellular messenger that regulates numerous functions in living organisms. Here, we describe development and characterization of ‘Salsa6f’, a fusion of GCaMP6f and tdTomato optimized for cell tracking while monitoring cytosolic Ca^2+^, and a transgenic Ca^2+^ reporter mouse with Salsa6f targeted to the Rosa26 locus for Cre-dependent expression in specific cell types. The development and function of T cells was unaffected in Cd4-Salsa6f mice. We describe Ca^2+^ signals reported by Salsa6f during T cell receptor activation in naive T cells, helper Th17 T cells and regulatory T cells, and Ca^2+^ signals mediated in T cells by an activator of mechanosensitive Piezo1 channels. Transgenic expression of Salsa6f enables ratiometric imaging of Ca^2+^ signals in complex tissue environments found in vivo. Two-photon imaging of migrating T cells in the steady-state lymph node revealed both cell-wide and localized sub-cellular Ca^2+^ transients (‘sparkles’) as cells migrate.

## eLife digest

To help protect the body from disease, small immune cells called T lymphocytes move rapidly, searching for signs of infection. These signs are antigens – processed pieces of proteins from invading bacteria and viruses – which are displayed on the surface of so-called antigen-presenting cells. To visit as many different antigen-presenting cells as possible, T cells move quickly from one to the next in an apparently random manner. How T cells are programmed to move in this way is largely unknown.

The entry of calcium ions into cells, through channel proteins, triggers characteristic actions in many cells throughout the body. As such it is possible that the T cells’ movements are related to calcium signals too. However, it was technically challenging to directly measure the amount of calcium in moving cells within the body.

To overcome this issue, Dong, Othy et al. genetically engineered mice to produce a new calcium-sensitive reporter protein in their T cells. The reporter, which was named Salsa6f, consisted of a red fluorescent protein fused to another protein that glows green when it binds to calcium ions. Measuring the ratio of red and green fluorescence gives a measure of the concentration of calcium ions inside the cell. In the absence of calcium signaling, the cells can still be tracked via the red fluorescence of Salsa6f. Importantly, the reporter did not affect the development or activity of the T cells in the mice. In a related study, Dong, Othy et al. then used their transgenic mice to ask whether calcium signals guide moving T cells as they search for antigens.

Future studies could use these transgenic mice to track the calcium ion concentration in numerous cell types. This would enable new approaches to relate the inner workings of cells to their behaviors in many different organ systems throughout the body.

## Introduction

Calcium (Ca^2+^) is an essential second messenger responsible for a wide variety of cellular functions (Berridge et al., 2000; Clapham, 2007; Berridge, 2012). Through the use of synthetic small molecule Ca^2+^ indicators such as fura-2 and fluo-4, imaging studies have greatly expanded our understanding of Ca^2+^ signaling dynamics (Tsien et al., 1982; Grynkiewicz et al., 1985). However, such indicators cannot be targeted to specific subcellular compartments or cell populations, and are unsuitable for long-term studies due to leakage out of cells. Moreover, they often do not faithfully report pure cytosolic Ca^2+^ signals, because of diffusion into cellular compartments such as the nucleus. One alternative to overcoming these limitations is with genetically encoded Ca^2+^ indicators (GECIs), first developed two decades ago as FRET-based fluorescence probes (Miyawaki et al., 1997; Romoser et al., 1997; Pérez Koldenkova and Nagai, 2013). Key advantages to GECIs include the capability for genetic targeting to specific cell types or subcellular organelles, measuring local Ca^2+^ levels by direct fusion to a protein of interest, modulation of expression levels by inclusion of an inducible promoter, and long-term studies due to continuous expression of the genetic indicator (Miyawaki et al., 1997; Pérez Koldenkova and Nagai, 2013). Despite these inherent advantages, the initial FRET-based GECI probes were not widely used as their performance fell far behind small molecule Ca^2+^ indicators, particularly in Ca^2+^ sensitivity, brightness, and dynamic range. Since then, successive rounds of design and contributions from multiple research groups have resulted in numerous variants of GECIs with high dynamic range and dramatically improved performance (Baird et al., 1999; Nakai et al., 2001; Tian et al., 2009; Zhao et al., 2011; Akerboom et al., 2012; Akerboom et al., 2013; Chen et al., 2013). Single fluorescent protein-based GECIs containing a circularly permutated green fluorescent protein (GFP) exhibit high brightness, fast response kinetics, and offer multiple color variants, including the GECO and the GCaMP series (Tian et al., 2009; Zhao et al., 2011; Akerboom et al., 2012; Chen et al., 2013). FRET-based GECIs have continued to evolve as well, with sequential improvements including incorporation of circularly permuted yellow fluorescent proteins (cpYFPs) to improve dynamic range in the yellow cameleon (YC) family (Nagai et al., 2004), use of troponin C as the Ca^2+^ sensing element in the TN indicator family (Heim and Griesbeck, 2004), computational redesign of the calmodulin-M13 interface to increase the range of Ca^2+^ sensitivity and reduce perturbation by native calmodulin in the DcpV family (Palmer et al., 2006), and complete redesign of the troponin C domain to increase response kinetics and reduce buffering of cytosolic Ca^2+^ in the TN-XXL family (Mank et al., 2006; Mank et al., 2008).

The latest generation of GECIs have crossed key performance thresholds previously set by small-molecule indicators, enabling GECIs to be widely applied in diverse Ca^2+^ imaging studies without sacrificing performance. Members of the GCaMP6 family are capable of tracking cytosolic Ca^2+^ changes from single neuronal action potentials, with higher sensitivity than small-molecule indicators such as OGB-1 (Chen et al., 2013). The availability of multicolored variants in the GECO family and the RCaMP series allowed for simultaneous measurement of Ca^2+^ dynamics in different cell populations in the same preparation, or in different subcellular compartments within the same cell (Zhao et al., 2011; Akerboom et al., 2013). These variants can be integrated with optogenetics to simultaneously evoke channel rhodopsin activity while monitoring localized Ca^2+^ responses in independent spectral channels (Akerboom et al., 2013). Moreover, individual GECIs can be tagged onto membrane Ca^2+^ channels to directly measure Ca^2+^ influx through the target channel of interest, enabling optical recording of single channel activity without the need for technique-intensive patch clamping (Dynes et al., 2016).

Another advantage of GECIs is their capability to be incorporated into transgenic organisms. Although several GECI-expressing transgenic mouse lines have already been reported, many of these studies used older variants of GECIs that are expressed only in selected tissues (Hasan et al., 2004; Ji et al., 2004; Tallini et al., 2006; Heim et al., 2007). The Ai38 mouse line overcomes these issues by combining GCaMP3 with a robust and flexible Cre/lox system for selective expression in specific cell populations (Zariwala et al., 2012). Based on a series of Cre-responder lines designed for characterization of the whole mouse brain (Madisen et al., 2010), the Ai38 mouse line contains GCaMP3 targeted to the Rosa26 locus but requires Cre recombinase for expression. By crossing Ai38 with various Cre mouse lines, GCaMP3 can be selectively expressed in specific cell populations. Thus, target cells may be endogenously labeled without invasive procedures, avoiding potential off-target side effects reported in GECI transgenic lines with global expression (Direnberger et al., 2012). The newly released PC::G5-tdT mouse line provides improved functionality by targeting a Cre-dependent GCaMP5G-IRES-tdTomato transgenic cassette to the *Polr2a* locus (Gee et al., 2014). However, in the PC::G5-tdT mouse line, GCaMP5G and tdTomato are expressed individually, and localize to different cell compartments. Moreover, because expression of tdTomato is driven by an internal ribosomal entry site, the expression level is highly variable and weaker than GCaMP5G, limiting identification of positive cells and preventing accurate ratiometric measurements.

Although single fluorescent protein-based indicators have high brightness and fast response kinetics, as non-ratiometric probes they are problematic for Ca^2+^ imaging in motile cells where fluorescence changes resulting from movement may be indistinguishable from actual changes in Ca^2+^ levels. Here, we introduce a novel genetically encoded Ca^2+^ indicator - that we christen ‘Salsa6f’ - by fusing green GCaMP6f to the Ca^2+^-insensitive red fluorescent protein tdTomato. This probe enables true ratiometric imaging, in conjunction with the high dynamic range of GCaMP6. We further describe the generation of a transgenic mouse enabling Salsa6f expression in a tissue-specific manner, and demonstrate its utility for imaging T lymphocytes in vitro and in vivo.

## Results

### A novel ratiometric genetically encoded Ca^2+^ indicator, Salsa6f

**Figure 1.**
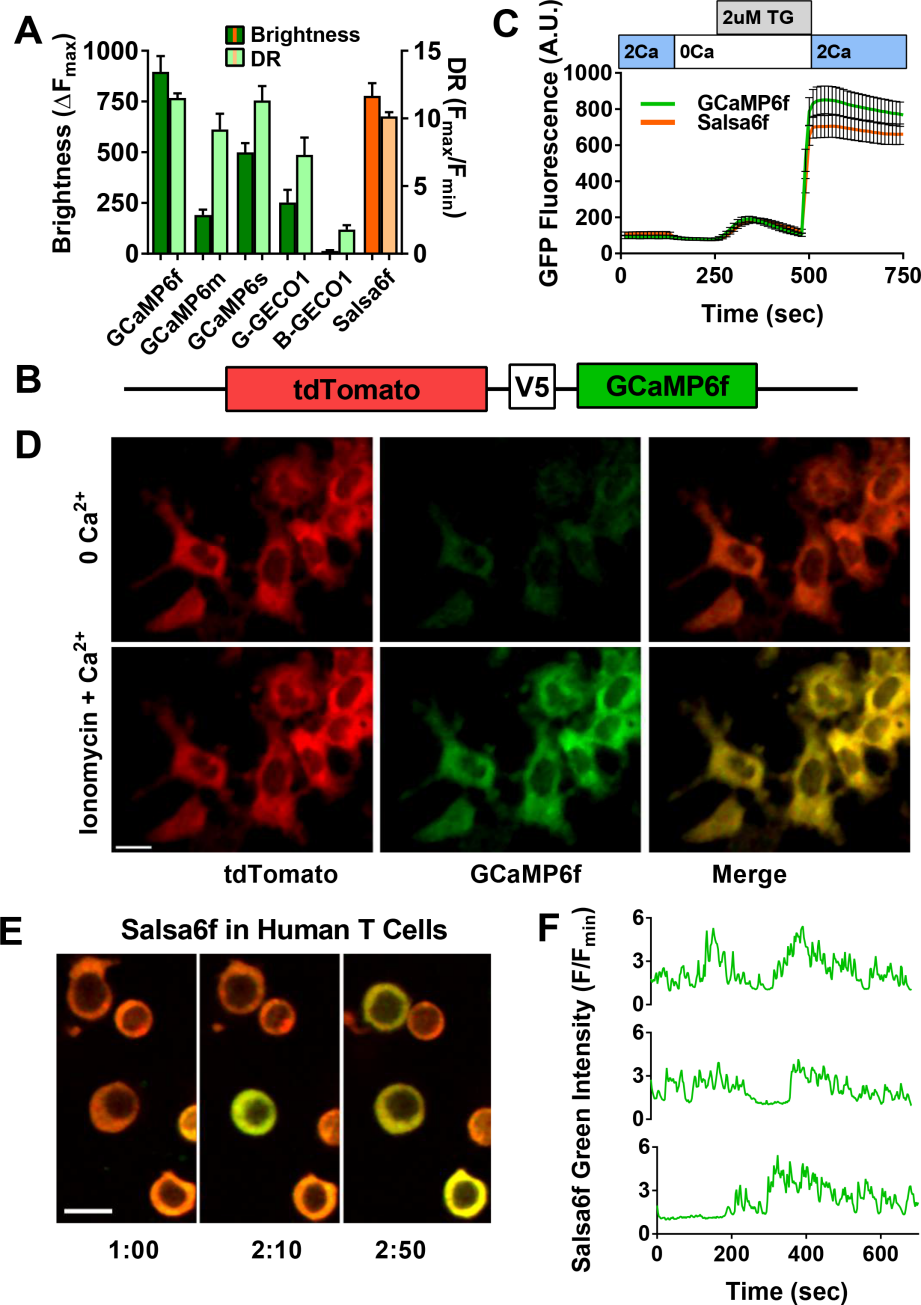
Design of novel tdTomato-V5-GCaMP6f fusion probe ‘Salsa6f’ and characterization in living cells. (**A**) Several genetically encoded Ca^2+^ indicators were screened in vitro in HEK 293A cells, by co-transfecting with Orai1/STIM1 and measuring Ca^2+^ influx after thapsigargin-induced store depletion. Bars indicate maximum change in fluorescence intensity (dark) and dynamic range (DR: light) with Salsa6f shown in orange bars on right; n > 30 cells per probe, from two different transfections, error bars indicate SEM. (**B**) Diagram of Salsa6f construct used in transfection. (**C**) Averaged thapsigargin-induced Ca^2+^ entry, measured by change in green fluorescence, in GCaMP6f- (green, 11.5 ± 0.3, n = 63) or Salsa6f- (orange, 10.2 ± 0.3, n = 78) transfected HEK cells; data from two different transfections, error bars indicate SEM. (**D**) Two-photon images of Salsa6f co-transfected in HEK cells with Orai1/STIM1, showing red (tdTomato), green (GCaMP6f), and merged channels, at baseline in 0 mM extracellular Ca^2+^ (top) and after maximum stimulation with 2 µM ionomycin in 2 mM extracellular Ca^2+^ (bottom); scale bar = 20 µm; see Video 1; data are representative of at least three different experiments. (**E**) Confocal time lapse microscopy of human Cd4^+^ T cells previously transfected with Salsa6f and then activated for 2 days on plate-bound αCd3/28 antibodies; time = min:s, scale bar = 10 µm. (**F**) Representative traces of green fluorescence intensity from individual activated human T cells transfected with Salsa6f. Data are representative of at least three different experiments.

In order to develop a better tool to monitor Ca^2+^ signaling in T cells both in vivo and in vitro, we first evaluated the latest generation of genetically encoded Ca^2+^ indicators (GECIs) (Zhao et al., 2011; Chen et al., 2013). We transiently expressed and screened a variety of single fluorescent protein-based GECIs in HEK 293A cells (Figure 1A), and selected GCaMP6f based on fluorescence intensity, dynamic range, and Ca^2+^ affinity suitable for detecting a spectrum of cytosolic Ca^2+^ signals (*K_d_* = 375 nM). To enable cell tracking even when basal Ca^2+^ levels evoke little GCaMP6f fluorescence, we fused GCaMP6f to the Ca^2+^-insensitive red fluorescent protein tdTomato, chosen for its photostability and efficient two-photon excitation (Drobizhev et al., 2011). A V5 epitope tag (Lobbestael E et al., 2010) served to link tdTomato to GCaMP6f (Figure 1B). The resultant ratiometric fusion indicator, coined ‘Salsa6f’ for the combination of red tdTomato with the green GCaMP6f, was readily expressed by transfection into HEK 293A cells and human T cells. Salsa6f exhibited a ten-fold dynamic range, with a brightness comparable to GCaMP6f alone (Figure 1A,C). For two-photon microscopy, both components of Salsa6f can be visualized by femtosecond excitation at 900 nm (Figure 1D). GCaMP6f produces increased green fluorescence during elevations in cytosolic Ca^2+^, while tdTomato provides a stable red fluorescence that facilitates cell tracking and allows for ratiometric Ca^2+^ imaging (Figure 1D; Video 1). Salsa6f is excluded from the nucleus, ensuring accurate measurement of cytosolic Ca^2+^ fluctuations (Figure 1D,E). When expressed by transfection in human T cells, Salsa6f reported Ca^2+^ oscillations induced by immobilized αCD3/28 antibodies with a high signal to noise ratio and time resolution (Figure 1E,F).

**Video 1.** Calcium readout of Salsa6f probe in HEK cells. HEK 293A cells transfected with Salsa6f, first washed with 0 mM Ca^2+^ followed by 2 μM ionomycin in 2 mM Ca^2+^; scale bar = 20 µm, time shown in hr:min:s. Images were acquired at 15 s interval and played back at 15 frames per second. This video corresponds to Figure 1D.

### Generation of Salsa6f transgenic reporter mice and validation in immune cells

**Figure 2.**
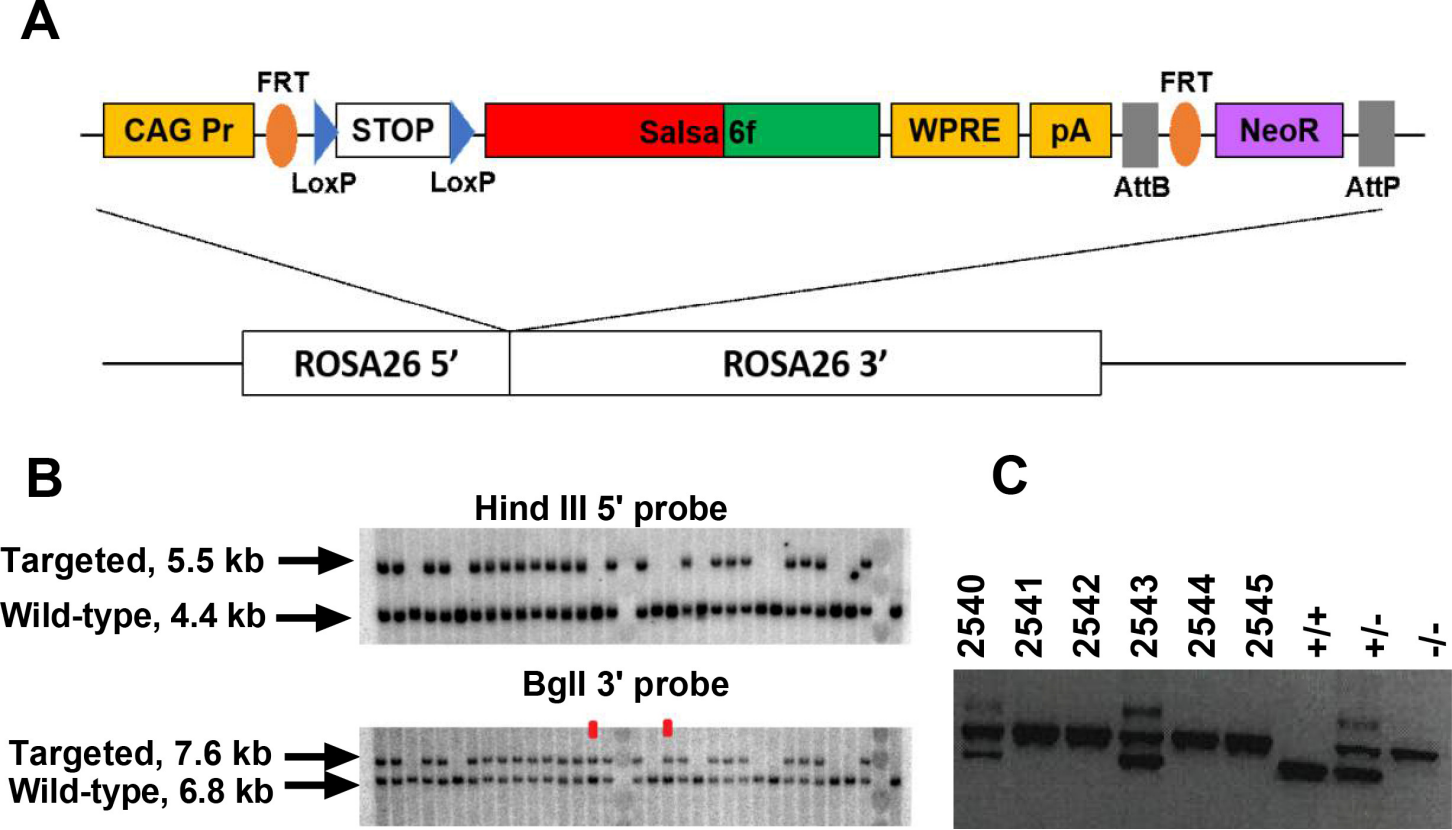
Generation of a Salsa6f transgenic mouse line targeted to the Rosa26 locus. (**A**) Transgenic targeting vector for Salsa6f, inserted between Rosa26 homology arms and electroporated into embryonic stem cells. CAG Pr: cytomegalovirus early enhancer/chicken β-actin promoter; Salsa6f: tdTomato-V5-GCaMP6f; FRT, LoxP, AttB, AttP: recombinase sites; WPRE: woodchuck hepatitis virus post-transcriptional regulatory element; pA: bovine growth hormone polyadenylation sequence; NeoR: neomycin resistance gene. (**B**) Correctly targeted ES cells were screened by Southern blot after HindIII digest for the 5’ end (top) or BglI digest for the 3’ end (bottom). The two clones marked in red failed to integrate at the 5’ end. (**C**) PCR screening for chimeras based on presence of the Nnt mutation, present only in JM8.N4 ES cells but not in the C57BL/6J blastocyst donors. 2540 and 2543 are chimeras. Control lanes on the right are wild type (*Nnt*^+^/^+^), heterozygous (*Nnt*^+/-^), or homozygous mutant (*Nnt*^-/-^).

Guided by the transgenic targeting strategy for the Ai38 mouse line (Zariwala et al., 2012), we inserted Salsa6f into a Gt(ROSA)26Sor5’-pCAG-FRT-LSL-Salsa6f-WPRE-bGHpA-AttB-FRT-NeoR-AttP-Gt(ROSA)26Sor3’ cassette, then targeted it to the Rosa26 locus in JM8.N4 mouse embryonic stem (ES) cells (Figure 2A). Cells positive for the allele *Gt(ROSA)26Sor^pCAG-FRT-LSL-Salsa6f-WPRE-bGHpA-AttB-FRT-NeoR-AttP^* were selected by neomycin resistance, and correctly targeted clones were screened by Southern blot (Figure 2B), then injected into C57BL/6J blastocysts for implantation. Chimeric pups carrying the Salsa6f transgene were identified by PCR screening for the *Nnt* gene, as the initial JM8.N4 ES cells were *Nnt*^+/+^ while the C57BL/6J blastocysts were *Nnt*^-/-^ (Figure 2C). Positive chimeras were bred to R26ΦC31o mice to remove the neomycin resistance gene and to produce LSL-Salsa6f F1 founders that are heterozygotic for the *Gt*(*ROSA*)*26Sor^pCAG-FRT-LSL-Salsa6f-WPRE-bGHpA-AttB/P^* allele, then further bred to generate homozygotic mice which we term LSL-Salsa6f (Hom).

**Figure 3.**
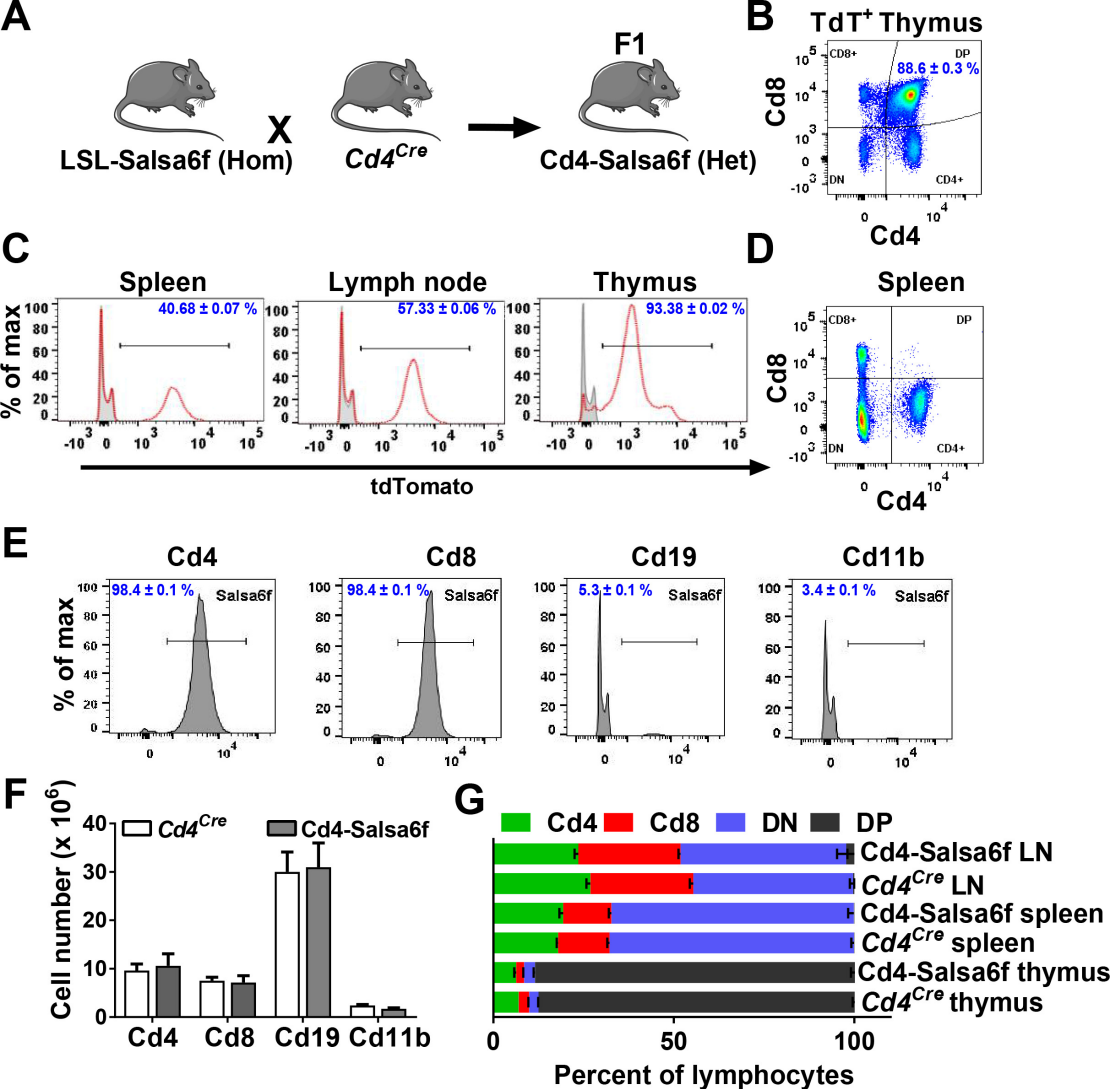
Cd4-Salsa6f mice show normal immune cell development and expression. (**A**) Experimental design to target expression of Salsa6f in Cd4 cells. (**B**) Cd4, Cd8 and double-positive cells gated on tdTomato (Salsa6f^+^ cells) from thymus. (**C**) Histograms showing percent of Salsa6f^+^ cells in spleen, LN, and thymus. (**D**) Cd4, Cd8, and double positive cells from spleen, gated on tdTomato (Salsa6f^+^ cells). (**E**) Histograms showing percent of Salsa6f^+^ cells within Cd4, Cd8, Cd19, Cd11b populations from spleen. (**F**) Total number of Cd4, Cd8, Cd19, Cd11b cells in the spleen of Cd4-Salsa6f (Het) mice and *Cd4^Cre^* mice (n = 6 mice). (**G**) Relative percentages of Cd4, Cd8, Cd19, Cd11b cells in thymus, lymph nodes, and spleen of Cd4-Salsa6f mice and *Cd4^Cre^* mice (n = 6).

LSL-Salsa6f (Hom) mice were bred to *Cd4^Cre^*^+/+^ mice to obtain reporter mice heterozygous for Salsa6f, designated as Cd4-Salsa6f (Het) mice from here on, that selectively express Salsa6f in T cells (Figure 3A). Mice homozygous for Salsa6f are designated as Cd4-Salsa6f (Hom). Salsa6f was detected by tdTomato fluorescence on flow cytometry. 88% of these Salsa6f^+^ cells in thymus were double positive for Cd4 and Cd8 (Figure 3B). This is due to the double-positive stage during development, in which developing thymocytes will express both Cd4 and Cd8 before undergoing positive and negative selection to become either mature Cd4^+^ or Cd8^+^ T cells. Salsa6f was readily detected by the red tdTomato signal in cells from spleen (40%), lymph node (57%), and thymus (93%) (Figure 3C). As expected, double positive cells were not detected in the spleen (Figure 3D). More than 98% of Cd4^+^ and Cd8^+^ T cells from these reporter mice were positive for Salsa6f. Salsa6f was also detected in 5% of Cd19^+^ cells and 3% of Cd11b^+^ cells (Figure 3E). A small fraction of B cells express Cd4 mRNA, which may explain the presence of Salsa6f in Cd19^+^ cells (Zhang and Henderson, 1994). Cd11b^+^ cells positive for Salsa6f may be splenic resident dendritic cells that also express Cd4 (Vremec et al., 2000; Turley et al., 2010). The total number and relative frequencies of Cd4^+^, Cd8^+^, Cd19^+^, and Cd11b^+^ cells were similar to the *Cd4^Cre^* controls (Figure 3F,G).

**Figure 4.**
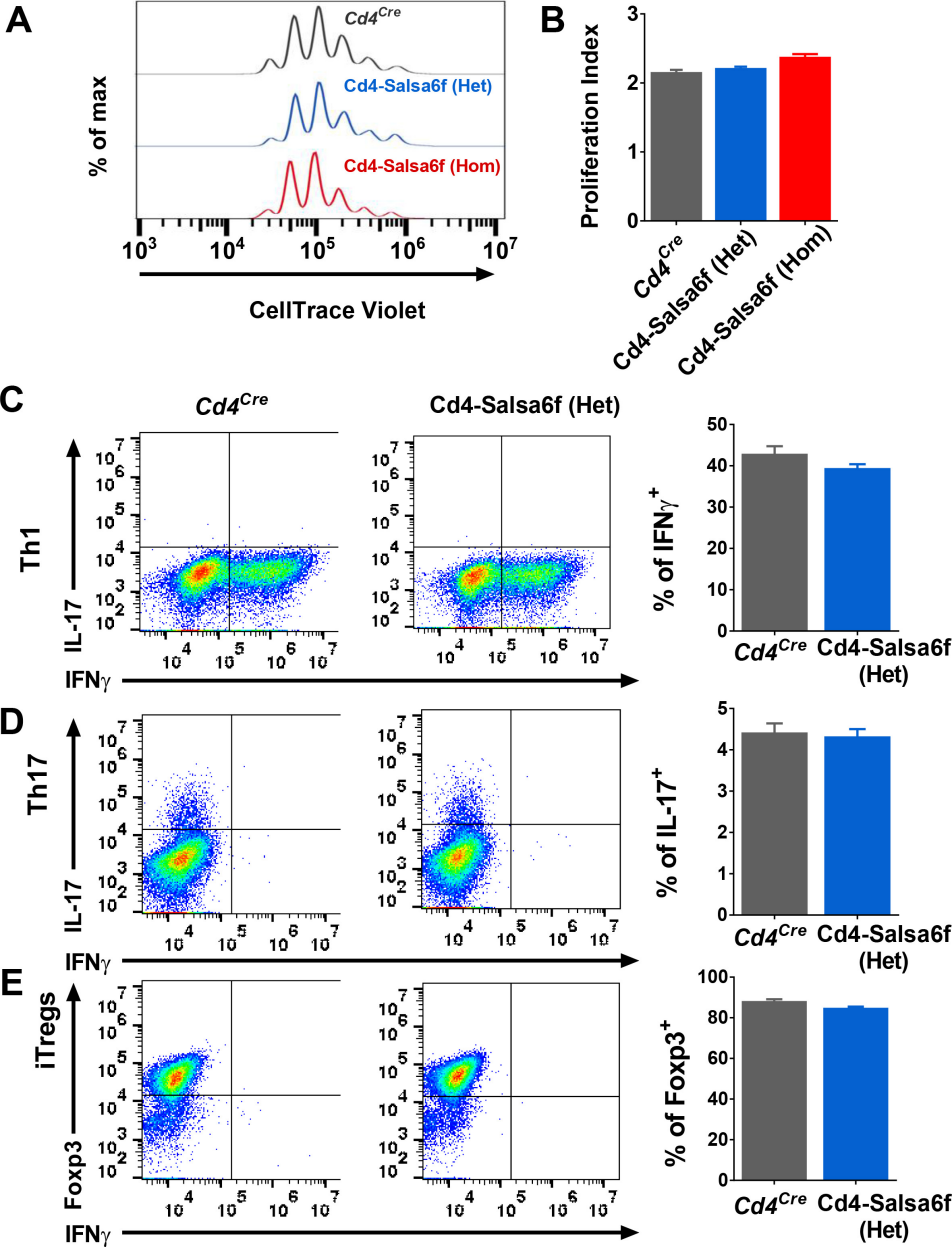
Functional responses of Cd4-Salsa6f T cells in vitro. (**A**) Representative histogram showing cell trace violet (CTV) dilution in *Cd4^Cre^* (dark grey), Cd4-Salsa6f (Het) (blue), and Cd4-Salsa6f (Hom) (red) T cells at 72 hr following stimulation with αCd3/28 Dynabeads (1:1 ratio). (**B**) Proliferation index measured on CTV dilution curves (n = 8). (**C–E**) Dot plots showing differentiation of naive T cells from *Cd4^Cre^* and Cd4-Salsa6f (Het) mice into Th1 cells (**C**), Th17 cells (**D**) and iTregs (**E**) after 6 days (n = 4 mice). Right panels show average percentages of IFNγ^+^ cells (**C**), IL-17^+^ cells (**D**) and Foxp3^+^ cells (**E**).

To determine whether expression of Salsa6f might affect functional responses downstream of Ca^2+^ signaling in T cells, we first purified Cd4^+^ T cells and monitored cell proliferation in vitro during TCR engagement of αCd3 and co-stimulating αCd28 antibodies attached to activating beads. Both hetero and homozygotic Salsa6f-expressing Cd4^+^ T cells proliferated similar to the *Cd4^Cre^* controls (Figure 4A,B). To further probe functional responses, we differentiated naive Cd4^+^ T cells using polarizing cytokine stimuli to generate Th1, Th17 and induced regulatory T cells (iTregs). Salsa6f-positive naive Cd4^+^ T cells from both Cd4-Salsa6f (Het) and Cd4-Salsa6f (Hom) mice readily differentiated into various helper T cell subtypes similar to the *Cd4^Cre^* controls (Figure 4C–E and Figure 4—figure supplement 1). In addition, as described in the companion paper, adoptively transferred Salsa6f-positive cells readily homed to lymph nodes and exhibited normal motility characteristics (Dong et al., 2017). In summary, our results demonstrate normal T-cell functions of Salsa6f-expressing T lymphocytes with respect to development, cellular phenotype, cell proliferation, differentiation, homing, and motility.

**Figure 4—figure supplement 1.**
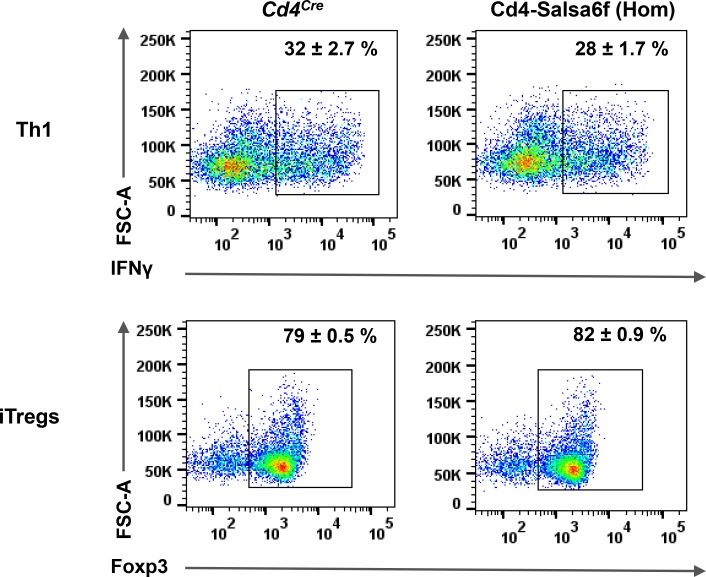
Differentiation of Cd4-Salsa6f (Hom) T cells into Th1 and iTregs. Representative dot plots showing differentiation of Cd4^+^ T cells from *Cd4^Cre^* and Cd4-Salsa6f (Hom) mice into Th1 cells (top panel) and iTregs (bottom panel) after 4 days of stimulation with αCd3/28 Dynabeads (1:1 ratio) in the presence of polarizing cytokines. Numbers inside the plots indicate percent of cells in respective gates (mean ±SD, n = 4).

### Single-cell ratiometric Ca^2+^ measurement in Cd4-Salsa6f reporter mice

**Figure 5.**
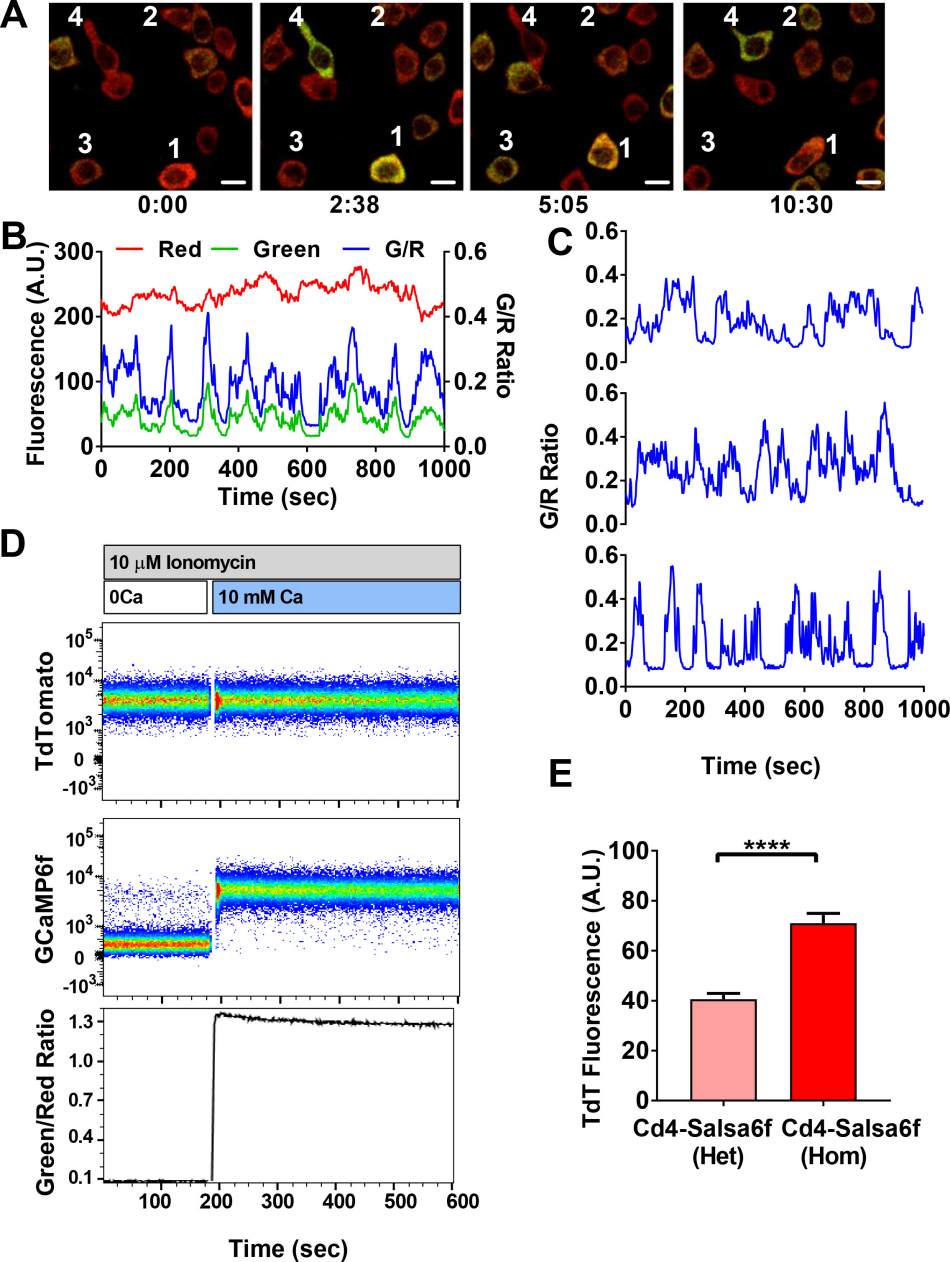
Single-cell Salsa6f calcium signals in T cells. (**A**) Confocal images of Ca^2+^ signals in activating Cd4^+^ T cells from Cd4-Salsa6f (Het) mice, after two day stimulation on plate bound αCd3/28 antibody, showing merged green (GCaMP6f) and red (tdTomato) channels; time = min:sec; scale bar = 10 μm. (**B**) Representative traces from cell #3 in (**A**), showing cell-wide fluorescence intensity changes in GCaMP6f (green), tdTomato (red), and green/red ratio (G/R, blue). (**C**) G/R ratios for cells 1, 2, and 4 from (**A**). (**D**) Dynamic range of Salsa6f in resting Cd4 T cells, measured as green/red fluorescence ratio by flow cytometry. Cells were pre-treated with 10 μM ionomycin in Ca^2+^-free solution (white bar), followed by re-addition of 10 mM Ca^2+^ (blue bar). (**E**) Averaged tdTomato fluorescence in resting T cells from heterozygous Cd4-Salsa6f compared to homozygotic Cd4-Salsa6f mice.

Cd4^+^ T cells were purified from Cd4-Salsa6f (Het) reporter mice, stimulated with plate-bound αCd3/28 antibodies for 2 days, and imaged by confocal microscopy while still in contact with immobilized antibodies (Figure 5A, Video 2). Activated Cd4^+^ T cells expressing Salsa6f exhibited stable red fluorescence and wide fluctuations in green fluorescence due to Ca^2+^ oscillations resulting from T-cell receptor engagement (Figure 5B). Despite variability in total fluorescence between cells due to individual differences in cell size, the basal and peak green/red Salsa6f ratios (referred from now on as G/R ratio for GCaMP6f/tdTomato intensity) were comparable between cells and showed up to six-fold increases during peaks in Ca^2+^ fluctuations (Figure 5C). Flow cytometric analysis of Salsa6f mouse T cells revealed a 13-fold increase in G/R ratio, by pretreatment with ionomycin in Ca^2+^-free medium to deplete cytosolic Ca^2+^ followed by addback of extracellular Ca^2+^, further emphasizing the high dynamic range of Salsa6f (Figure 5D). Finally, to test if increasing the genetic dosage can improve the brightness of Salsa6f, we compared Cd4^+^ T cells from Cd4-Salsa6f (Het) and Cd4-Salsa6f (Hom) mice. T cells from homozygous mice with two allelic copies of the Salsa6f reporter cassette exhibited almost a two-fold increase in tdTomato fluorescence compared to heterozygous mice (Figure 5E), allowing for genetic control of Salsa6f expression level when brightness is an issue.

**Video 2.** Single-cell readout of activation in transgenic T cells by Salsa6f. Cd4 T cells from Cd4-Salsa6f (Het) mice were plated on activating surface coated with anti-Cd3/Cd28. Images were acquired at 5 s interval and played back at 15 frames per second. This video corresponds to Figure 5A.

### Cytosolic localization and calibration of Salsa6f in transgenic T lymphocytes

**Figure 6.**
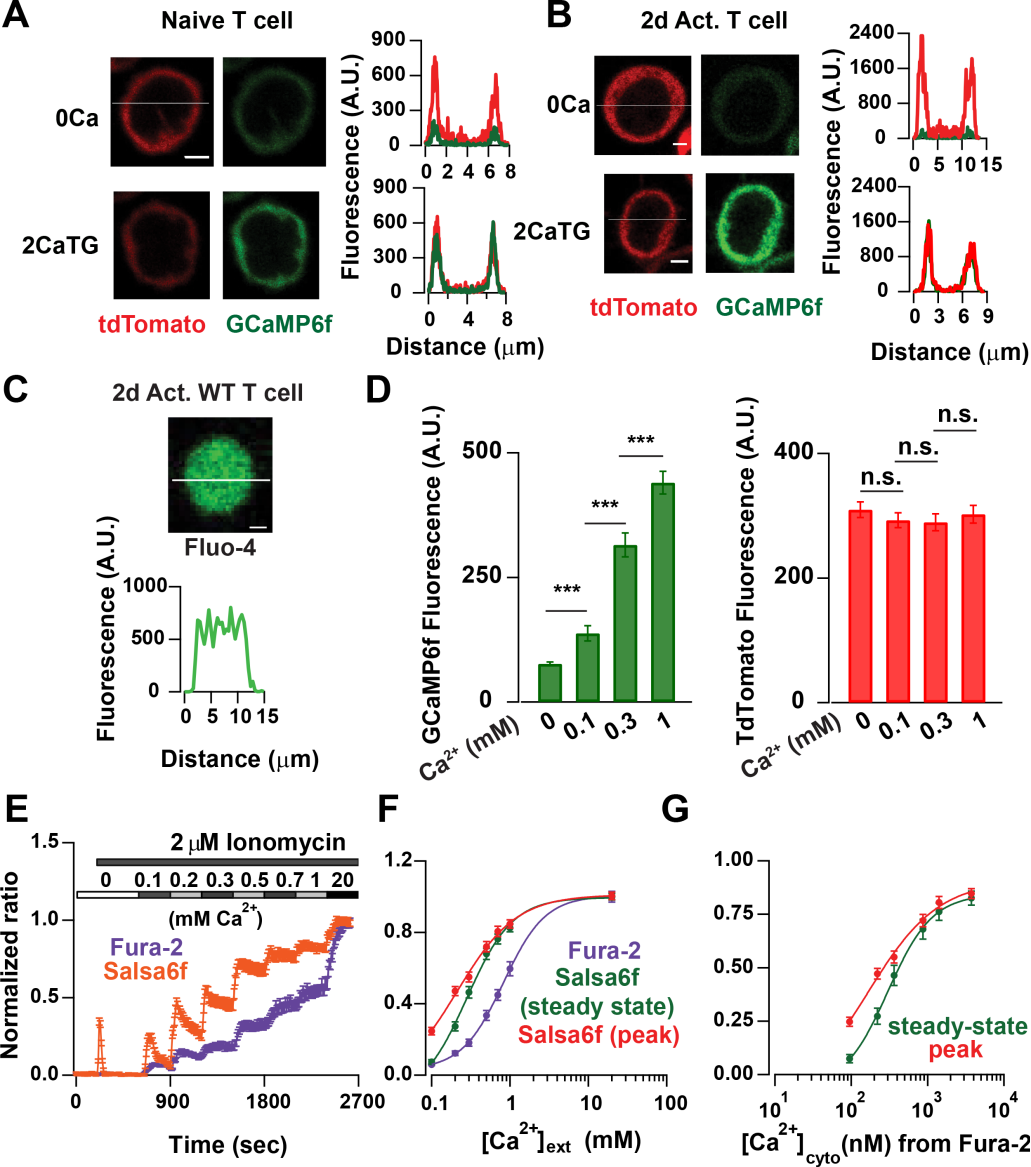
Probe characterization and calibration of [Ca^2+^] in Salsa6f T cells. (**A**) Confocal images of a naive T cell from a Cd4-Salsa6f (Het) mouse. Upper panel: tdTomato (left) and GCaMP6f (right) fluorescence intensity in Ca^2+^-free Ringer solution. Lower panel: same cell treated with 2 μM thapsigargin (TG) in Ringer solution containing 2 mM Ca^2+^. Line scans for each condition are shown adjacent to the images. Scale bar = 2 μm for **A–C**). (**B**) Corresponding confocal images and line scans of Salsa6f localization in a 2-day activated Cd4^+^ T-cell from Cd4-Salsa6f (Het) mouse. (**C**) Confocal image of a Fluo-4 (5 μM)-loaded Cd4^+^ T cell from *Cd4^Cre^* mouse. (**D**) Average GCaMP6f and tdTomato intensities in 2-day activated Cd4^+^ T cells treated with 2 μM ionomycin in Ca^2+^-free buffer and in external Ringer solution containing 0.1, 0.3 and 1 mM Ca^2+^. n = 36 cells, representative of three experiments. (**E**) Average 340/380 nm ratios in 2-day-activated and fura-2-loaded Cd4^+^ T cells from *Cd4^Cre^* mice (n = 59 cells) and G/R ratios in 2-day activated Cd4^+^ T cells from Cd4-Slasa6f (Het) mice (n = 47 cells) treated identically with 2 μM ionomycin followed by graded increases of external Ca^2+^ concentration as indicated. (**F**) Steady-state fura-2 and Salsa6f ratios recorded 300 s after solution application and peak Salsa6f ratio from **6E** plotted as a function of external Ca^2+^ concentration. (**G**) Steady-state and peak Salsa6f ratios plotted as a function of cytosolic Ca^2+^ concentrations calculated from the fura-2 experiment, assuming a fura-2 *K_d_* of 225 nM. The points were fit with a four parameter Hill equation to obtain the *K_d_* for Salsa6f, with the following parameters: Salsa6f steady-state: Hill coefficient = 1.49 ± 0.16; *K_d_* = 301 ± 24; Salsa6f peak: Hill coefficient = 0.93 ± 0.4; *K_d_* = 162 ± 48. Data are representative of three experiments.

We first examined the localization of Salsa6f in naïve Cd4^+^ T cells isolated from Cd4- Salsa6f (Het) mice and in Cd4^+^ T cells activated for 2 days on plate-bound αCd3/28. Line scans of the confocal images of cells plated on poly-L-lysine-coated coverslips showed that Salsa6f is primarily localized to the cytoplasm and is excluded from the nucleus (Figure 6A,B). Increasing the cytosolic Ca^2+^ levels using thapsigargin (TG) in 2 mM Ca^2+^ Ringer’s solution caused a selective increase in the GCaMP6f signal, without altering the localization of Salsa6f probe. In contrast, the small-molecule dye Ca^2+^ indicators fluo-4 or fura-2 loaded into Cd4^+^ T cells from *Cd4^Cre^* mice are distributed throughout the cell, including the nucleus (Figure 6C and data not shown). A different transgenic mouse, PC::G5-tdT utilizes an internal ribosomal entry site to express both tdTomato and GCaMP5G as separate proteins that localize differently (Gee et al., 2014), with tdTomato distributed throughout the cell including the nucleus and GCaMP5G predominantly in the cytosol (Figure 6—figure supplement 1). In contrast, our tandem probe, Salsa6f, results in both red and green fluorescent proteins co-localized in the cytosol, allowing true ratiometric Ca^2+^ imaging and facilitating tracking of cells.

**Figure 6—figure supplement 1.**
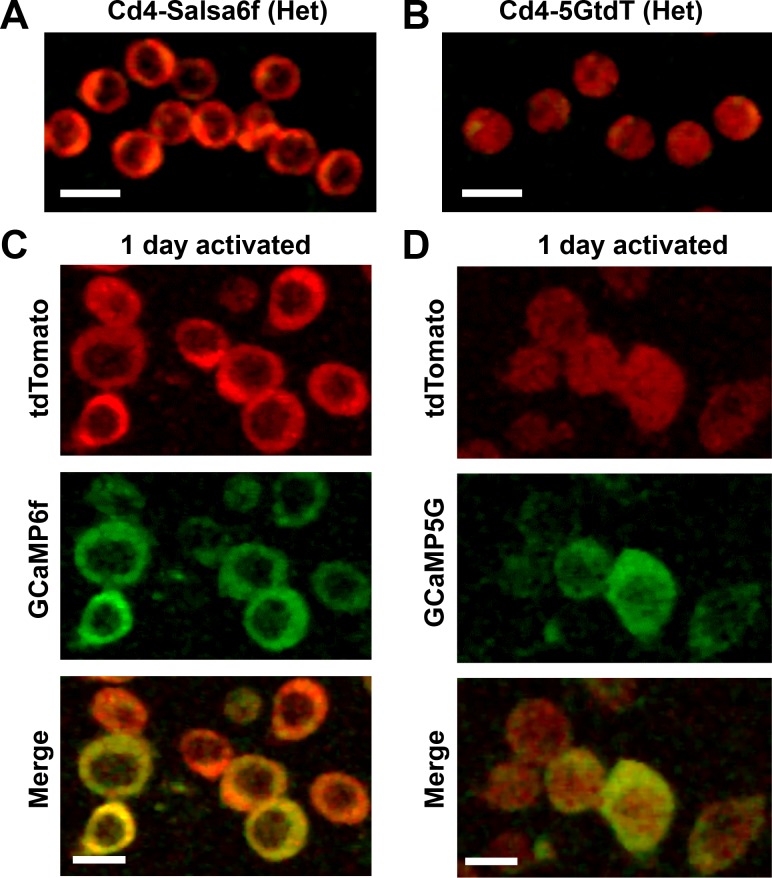
Comparison of GECI localization in Cd4 T cells from Salsa6f mouse and PC::G5-tdT mouse. (**A,B**) Confocal images of Cd4^+^ T cells purified from a Cd4-Salsa6f (Het) mouse (**A**) or a Cd4*-*5GtdT mouse (**B**), showing merged red and green; cells imaged at the same laser and PMT settings; scale bar = 10 μm. (**C,D**) Cd4^+^ T cells purified from a Cd4-Salsa6f (Het) mouse (**C**) or a Cd4*-*5GtdT mouse (**D**), then activated for 24 hr on plate-bound αCd3/28 antibodies, and imaged with confocal microscopy, showing red (tdTomato), green (GCaMP6f or GCaMP5G), and merged channels; scale bar = 10 μm.

Overlap of GCaMP6f emission and tdTomato excitation spectra raises the possibility of FRET (Forster resonance energy transfer) which would decrease G/R ratio and reduce dynamic range. If there were significant FRET, we would expect acceptor (tdTomato) signal to increase with increase in donor (GCaMP6f) intensity, as shown for GECI probes utilizing GCaMP and mCherry dependent on the rigidity of the linker used (Cho et al., 2017). We treated Salsa6f-positive T cells with Ringer’s buffer containing Ionomycin and different concentrations of Ca^2+^ and measured the steady-state GCaMP6f and tdTomato fluorescence signals. The GCaMP6f signal increased as expected with increasing Ca^2+^ concentrations, but the tdTomato signal remained unchanged (Figure 6D). This suggests that the V5 epitope tag is effective in reducing FRET between GCaMP6f and tdTomato which otherwise may compromise probe performance.

We next characterized the in situ Ca^2+^ affinity of Salsa6f in Cd4^+^ T cells isolated from Cd4-Salsa6f (Het) mice, using fura-2 as a calibration standard. Fura-2 has an in situ *K_d_* ~225 nM at 25° C (Lewis and Cahalan, 1989), which is somewhat lower than in vitro *K_d_* values reported for GCaMP6f (Chen et al., 2013; Badura et al., 2014). We used time-lapse imaging to record G/R ratio in response to ionomycin applied with stepwise increases in the external Ca^2+^ concentration and, using identical protocols, compared it to fura-2 Ca^2+^ signals in control Cd4^+^ T cells from *Cd4^Cre^* mice (Figure 6E,F). To facilitate comparison, fura-2 and Salsa6f ratios were normalized with R_min_ = 0 and R_max_ = 1. Salsa6f responded with faster rise and decay kinetics than fura-2 to progressive increases in cytosolic Ca^2+^ levels, especially at lower external Ca^2+^ concentrations. Salsa6f responses also saturated at lower cytosolic Ca^2+^ levels than fura-2 responses. This is not altogether surprising given that genetically encoded Ca^2+^ indicators have been reported to have a steeper Hill coefficient than chemical indicators (Badura et al., 2014). Assuming that Cd4-Salsa6f T cells reach similar Ca^2+^ levels as control *Cd4^Cre^* cells, we plotted peak and the steady state G/R ratios against the cytosolic Ca^2+^ concentrations obtained from the fura-2 experiment which yielded an estimated in situ *K_d_* for Salsa6f in the range of 160–300 nM (Figure 6G). Based on these results, we conclude that Salsa6f is sensitive in detecting cytosolic Ca^2+^ from 100 nM - 2 μM, which is in the range of physiological cytosolic Ca^2+^ signals.

### T-cell Ca^2+^ signaling in response to Ca^2+^ store depletion, T-cell receptor engagement, and mechanical stimulation

TCR engagement activates a canonical Ca^2+^ signaling pathway in T cells, characterized by IP_3_-induced Ca^2+^ release from the endoplasmic reticulum, leading to store-operated Ca^2+^ entry (SOCE) through Orai1 channels (Cahalan and Chandy, 2009; Prakriya and Lewis, 2015). Past studies on T cell Ca^2+^ signaling have largely relied on indicators like fura-2 and fluo-4 which have the potential drawback of being distributed into the nucleus, thus confounding the measurement of pure cytoplasmic Ca^2+^ signals, a problem particularly notable in T cells with their large nuclear to cytoplasmic volume ratio. Salsa6f, with its high dynamic range, ratiometric readout and targeted localization in the cytosol, is thus well suited to record physiological cytosolic Ca^2+^ signals. To this end, we recorded Ca^2+^ signals from 2-day-activated Cd4^+^ T cells from Cd4-Salsa6f (Het) mice in response to a variety of stimuli.

**Figure 7.**
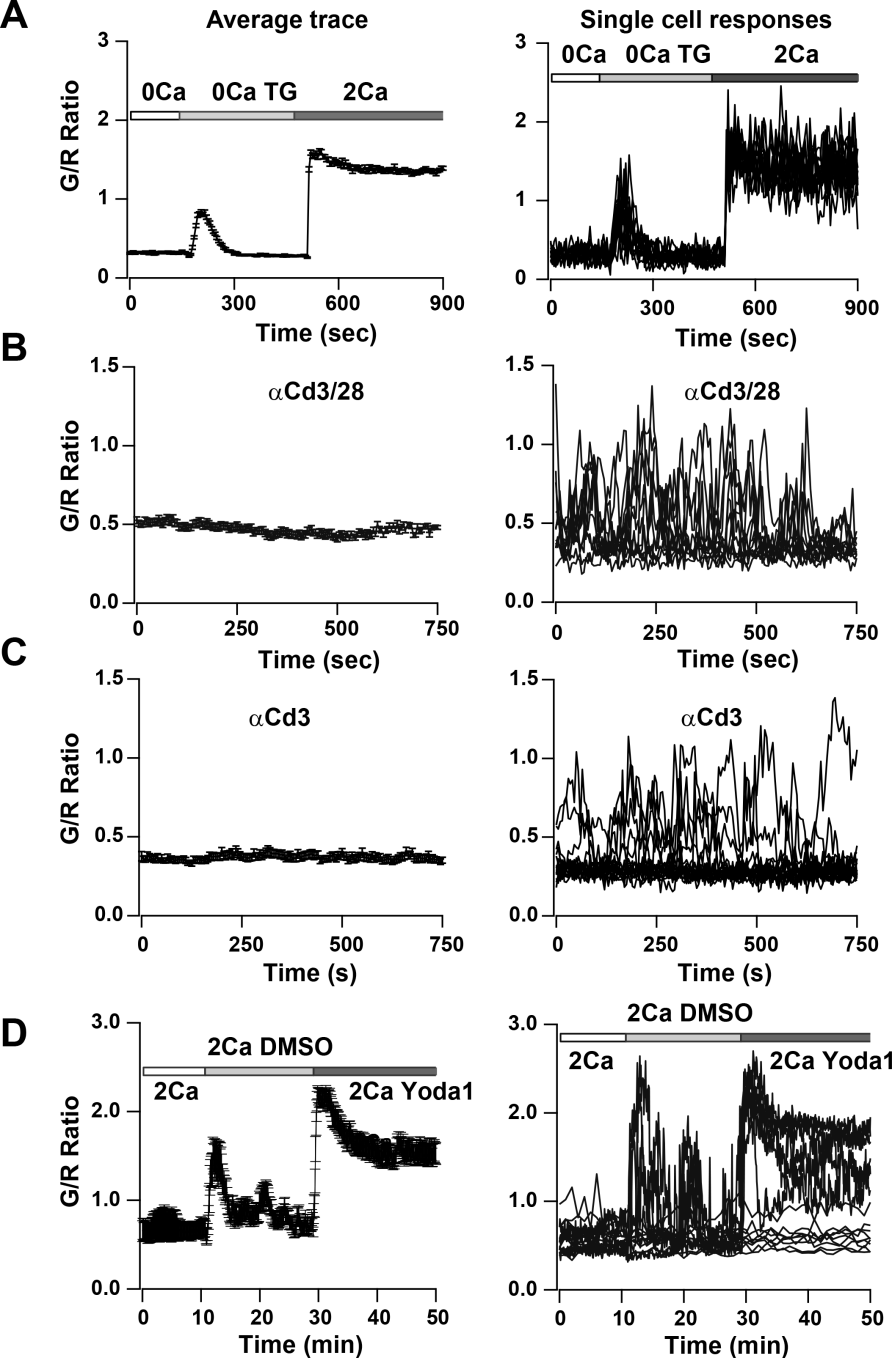
Ca^2+^signals in two day activated Cd4^+^ T cells from Cd4-Salsa6f (Het) mice in response to store-depletion, TCR stimulation and stimulation by Yoda1. In all panels, average Salsa6f G/R ratios are shown on the left, and representative single-cell traces are shown superimposed on right. Experiments were done in standard Ringer solution (**A**) or in RPMI containing 2% FCS and 2 mM Ca^2+^ (**B–D**). (**A**) Store-operated Ca^2+^ entry (SOCE) in Cd4^+^ T cells (n = 86 cells), induced by depleting ER Ca^2+^ stores with TG in Ca^2+^-free buffer followed by re-addition of Ringer containing 2 mM Ca^2+^. (**B,C**) Ca^2+^ responses to TCR stimulation in T cells plated on coverslips coated with 1 μg/ml αCd3/Cd28 (**B**) or 1 μg/ml αCd3 alone (**C**) (n = 90 cells each). (**D**) Ca^2+^ elevations during shear stress induced by solution exchange followed by the Piezo1 agonist Yoda1 (15 μM) in cells plated on αCd3/28 (n = 79 cells).

To study SOCE more directly, we depleted ER Ca^2+^ stores in activated and in naïve T cells by applying TG in Ca^2+^-free solution. We observed a small but sharp initial peak indicating ER store release followed by a sustained Ca^2+^ signal upon restoring Ca^2+^ to the external bath, indicative of SOCE (Figure 7A, Video 3 and Figure 7—figure supplement 1A). Almost all cells responded to this supra-physiological stimulus (Figure 7A, right panel). In contrast, T cells plated on αCd3/28 to activate TCR-induced signaling showed heterogeneous responses, with some exhibiting asynchronous Ca^2+^ oscillations of varying frequencies and durations whereas others failed to respond (Figure 7B, Video 4). Past studies have attributed these Ca^2+^ oscillations to SOCE from repetitive opening and closing of Orai1 channels allowing Ca^2+^ to enter T cells in a periodic and asynchronous manner (Lewis and Cahalan, 1989; Dolmetsch and Lewis, 1994), unlike the sustained activation with TG treatment. Cells plated on αCd3 alone also showed rhythmic oscillatory Ca^2+^ signals; however, the percentage of responding cells was significantly lower than with αCd3/28, resulting in a lower average signal compared with αCd3/28 stimulation (Figure 7C, Video 5) These results suggest that co-stimulatory signaling through Cd28 enhances the response to TCR activated Ca^2+^ signals, in alignment with previous observations (Chen and Flies, 2013).

**Figure 7—figure supplement 1.**
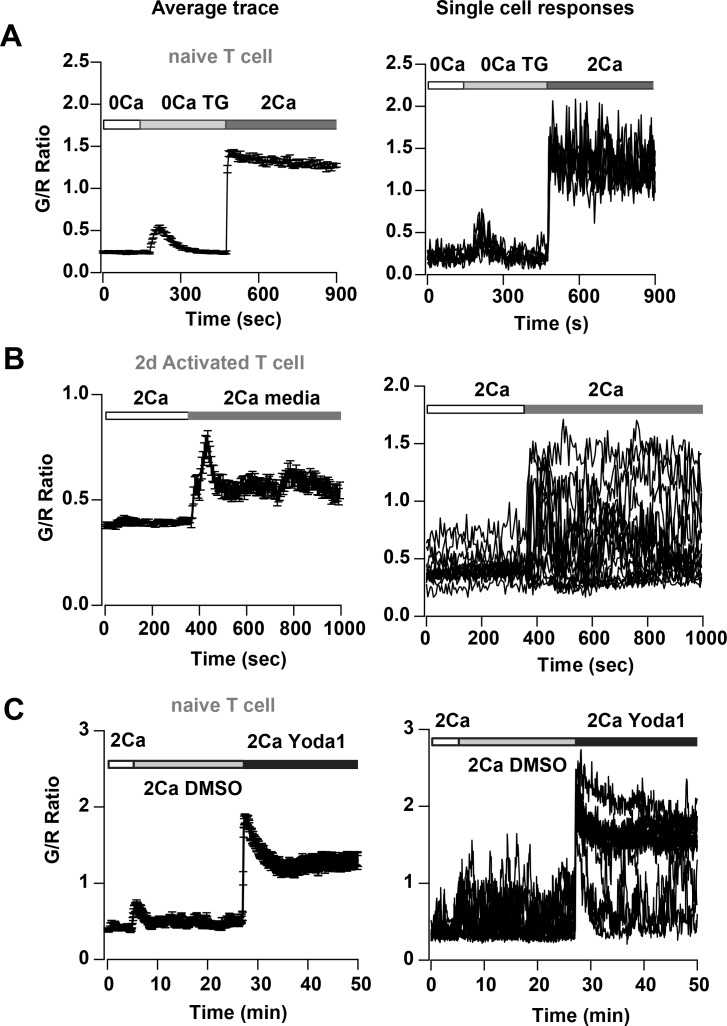
Store-operated Ca^2+^entry and Piezo1 activation in T cells from Cd4-Salsa6f (Het) mice. Average (left panel) and representative single-cell responses (right panel) to (**A**) TG-induced SOCE in naive T cells (n = 96 cells), (**B**) solution exchange with media alone in 2-day activated T-cells (n = 68 cells) and (**C**) application of 15 μM Yoda1 in naïve T cells (n = 53 cells).

**Video 3.** T cell Ca^2+^response to Ca^2+^store depletion by thapsigargin (TG). Video of maximum intensity projection images of 2-day activated T cells from Cd4-Salsa6f (Het) mouse plated on poly-L-lysine. Scale bar = 20 µm, time shown in hr:min:s. 2 μM TG in Ca^2+^-free Ringer’s was added at 00:02:30 and 2 mM Ca^2+^was added at 00:08:15. Time interval between frames is 5 s. Play back speed = 50 frames per second. This video corresponds to Figure 7A.

**Video 4.** Activated T cell Ca^2+^responses to TCR stimulation. Video of maximum intensity projection images of 2-day activated T cells from Cd4-Salsa6f (Het) mouse plated on anti-Cd3/28-coated coverslip. Scale bar = 20 µm, time shown in hr:min:s. Time interval between frames is 5 s. Play back speed = 15 frames per second. Video corresponds to Figure 7B.

**Video 5.** T cell Ca^2+^response to shear and Yoda1. Video of maximum intensity projection images of 2-day activated T cells from Cd4-Salsa6f (Het) mouse plated on anti-Cd3/28-coated coverslip. Scale bar = 20 µm, time shown in hr:min:s. Time interval between frames is 5 s. Play back speed = 200 frames per second. Medium was added at 00:15:00and Yoda1 was added at 00:35:00. Video corresponds to Figure 7C.

Finally, we focused on a novel Ca^2+^ signaling pathway involving mechanosensitive Ca^2+^-permeable channels. We examined activation of mechanosensitive channels in Salsa6f^+^ T cells that were plated on αCd3/28-coated glass coverslips, by flowing external solution rapidly past the cells. Perfusion produced a transient rise in the cytosolic Ca^2+^ signal, likely a result of flow shear stress (Figure 7D and Figure 7—figure supplement 1B). The Piezo family of mechanosensitive channels plays a vital role in cell motility and development (Nourse and Pathak, 2017), but it is not known whether these channels are expressed and play a role in immune cell function. Perfusion of medium including Yoda1, a selective small molecule activator of Piezo1 (Syeda et al., 2015), resulted in robust Ca^2+^ signals in activated and naive T cells that were larger and more sustained than those activated by perfusion of solvent or media alone (Figure 7D and Figure 7—figure supplement 1C). In summary, we show Ca^2+^ signals in T cells in response to an activator of Piezo1 channels. These results illustrate the utility of Salsa6f for screening agents that modulate Ca^2+^ signaling in T cells and open the possibility for further exploration of the functional role of Piezo1 channels in T cell function.

### TCR-induced Ca^2+^ signaling in helper T cell subsets

**Figure 8.**
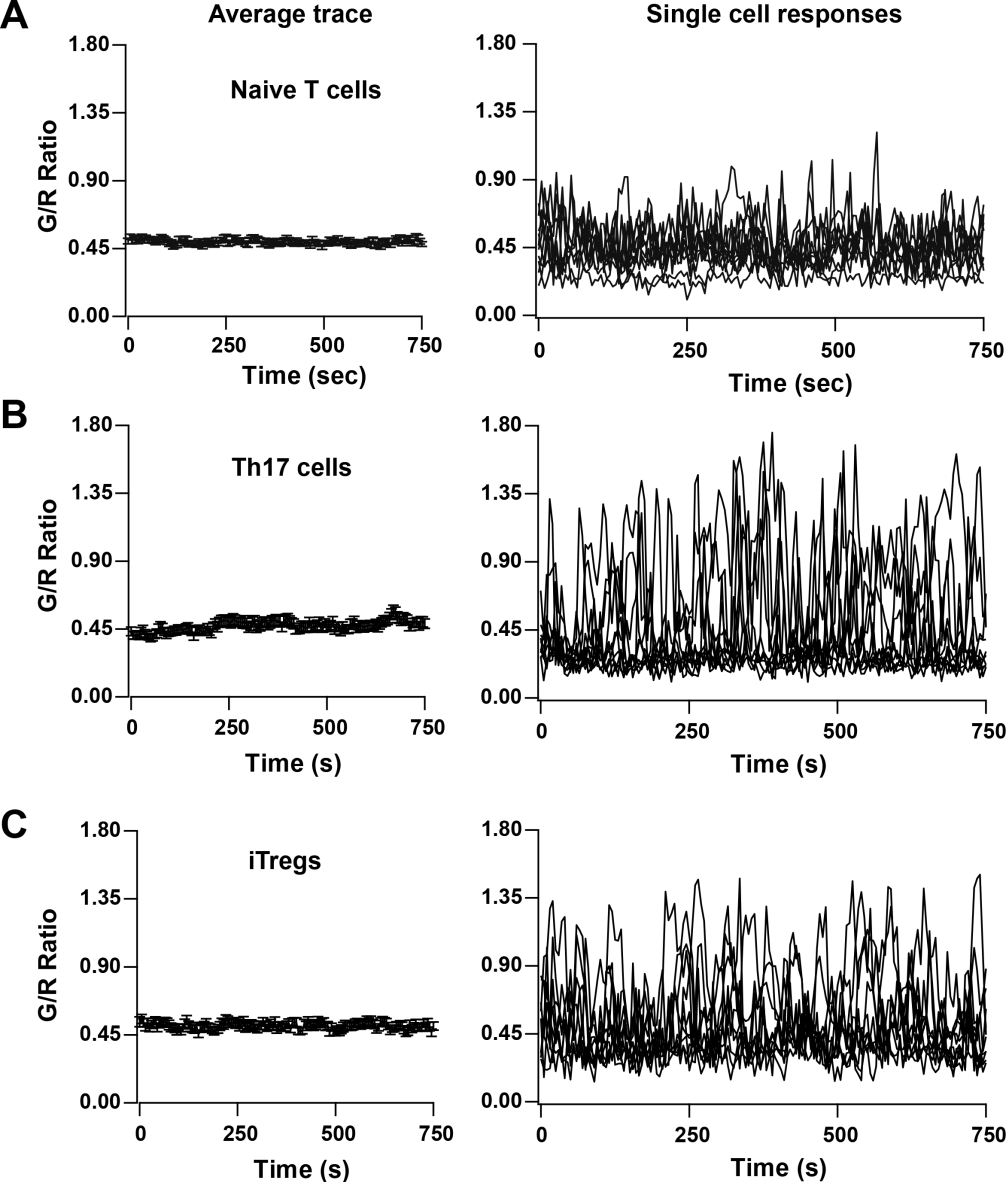
TCR induced Ca^2+ ^signals in T cell subsets from Cd4-Salsa6f (Het) mice. Average (left) and representative single-cell Ca^2+^ traces (right) from confocal time-lapse microscopy showing changes in Salsa6f green/red (G/R) ratio in naive T cells (**A**), 5-day differentiated Th17 cells (**B**), and 5-day differentiated iTregs (**C**) plated on 1 μg/mL αCd3/28. (n = 90 cells from two to three experiments each).

We also monitored Ca^2+^ signaling in response to TCR activation by αCd3/28 in various subsets of T cells from Cd4-Salsa6f (Het) mice, including naive T cells, Th17 cells and iTregs (Figure 8A–C). All subtypes of T cells responded to plate-bound stimulation of αCd3/28 with oscillatory changes in their cytosolic Ca^2+^ levels, very similar to the Ca^2+^ responses in 2-day-activated T cells illustrated in Figure 7B. The responses were heterogeneous, with some cells showing multiple peaks of varying durations and amplitudes, occasional sustained signals and other cells failing to respond. Whereas the overall average responses were not very different between the three subtypes examined, some individual Th17 cells and iTregs showed higher amplitude signals than any naive T cells, but with a greater percentage of non-responding cells. The Cd4-Salsa6f mouse thus opens up new avenues to study the fundamental nature of Ca^2+^ signals in T cell subsets generated in response to variety of stimuli, and to explore the relationship between patterns of Ca^2+^ signals and specific downstream functions.

### Two-photon microscopy of Cd4-Salsa6f T cells in the lymph node

**Figure 9.**
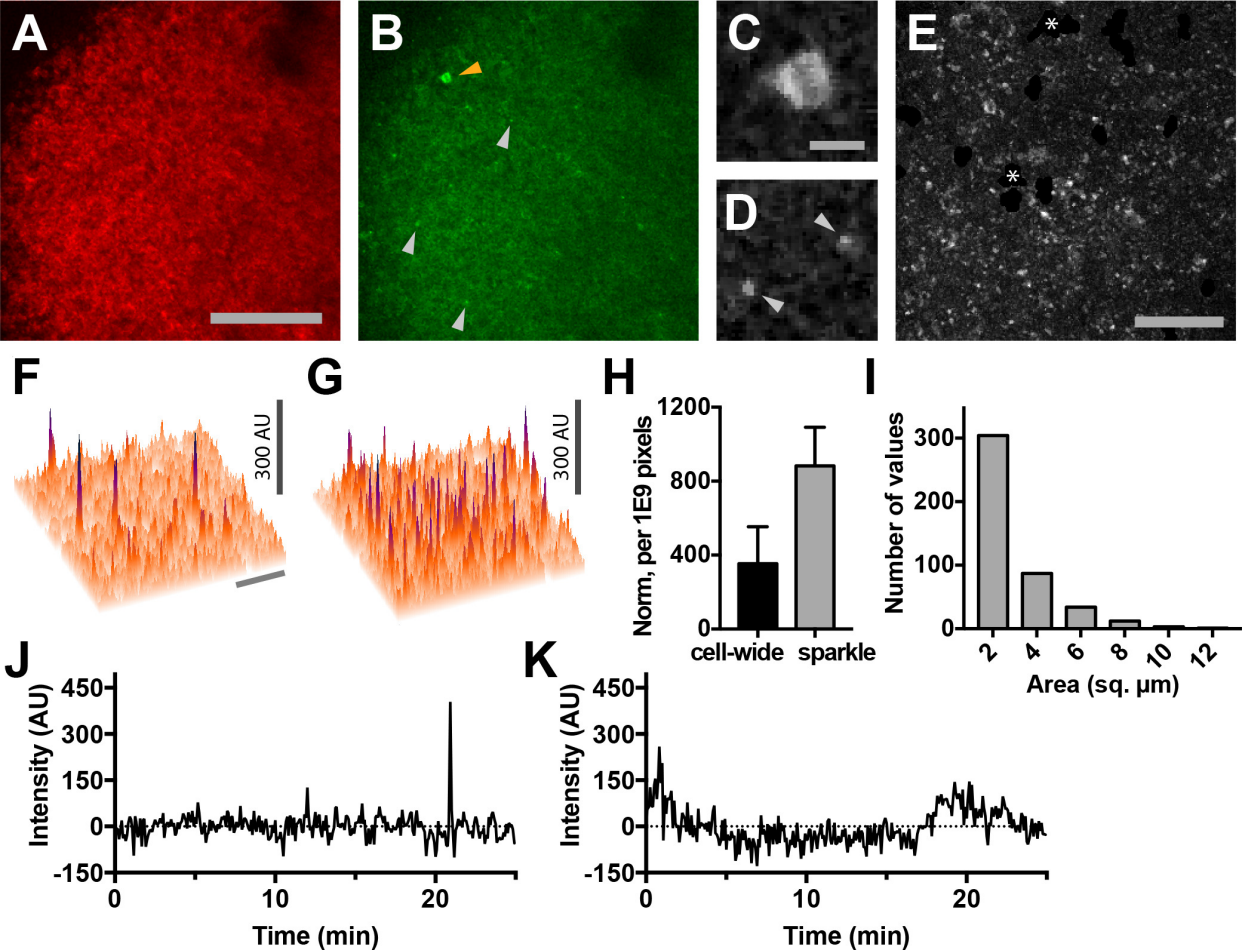
Lymph nodes from Cd4-Salsa6f (Hom) mice exhibit cell-wide and subcellular Ca^2+^signals. (**A**) Median filtered, maximum intensity projection of a red channel image from a single time point of an explanted lymph node from a Cd4-Salsa6f (Hom) mouse. (**B**) Green channel image corresponding to **A**). Orange arrowhead indicates cell-wide Ca^2+^ signal and gray arrowheads indicate smaller, local transient Ca^2+^ signals. (**C, D**) Enlargements of cell-wide (**C**) and local (**D**; gray arrowheads) Ca^2+^ signals. Note the lower fluorescence intensity in the center of the cell in C due to exclusion of Salsa6f from the nucleus. (**E**) Maximum intensity projection of 214 green channel time points (every 11.5 s over 41 min) showing hundreds of small local Ca^2+^ signals. Green channel image series was red channel subtracted and cropped from **B**. Asterisks indicate regions containing autofluorescent cells that have been cropped out. (**F, G**) Surface plot of maximum green channel intensity over two (**F**) and 50 (**G**) consecutive time points. Note the presence of four (**F**) and dozens (**G**) of small, discrete, high-intensity peaks of similar intensity. (**H**) Bar graph of relative frequencies of cell-wide and local Ca^2+^ signals. (**I**) Frequency distribution of the area of local Ca^2+^ signals. Scale bar in **A** is 100 µm (applies to **B**); scale bar in **C** is 10 µm (applies to **D**), scale bars in **E** and in **F** are 50 µm (applies to **G**). (**J**) Trace of fluorescence intensity over 25 min at the location of a transient subcellular Ca^2+^ signal (one time point every 5 s). (**K**) Trace of fluorescence intensity of a putative cell process from an autofluorescent cell drifting in the image field.

Using two-photon microscopy to image lymph nodes from Cd4-Salsa6f (Hom) mice under steady-state conditions, we observed sporadic localized elevations of green fluorescence indicative of intracellular Ca^2+^ signaling (Figure 9A,B). Imaging conditions were optimized for single-wavelength excitation of both TdTomato and GCaMP6f components of Salsa6f (Figure 9—figure supplement 1). Some of the green Salsa6f signals were T cell-sized, and the pattern of red Salsa6f fluorescence observed is consistent with the exclusion of the Salsa6f indicator from the nucleus (Figure 9C). In addition, we observed numerous bright, transient green fluorescent signals which were much smaller in area (about 2 µm^2^ in area) (Figure 9B,D; Video 6). We term these fluorescent transients ‘sparkles’, because during rapid playback of time-lapse image streams cells appear to sparkle.

**Figure 9—figure supplement 1.**
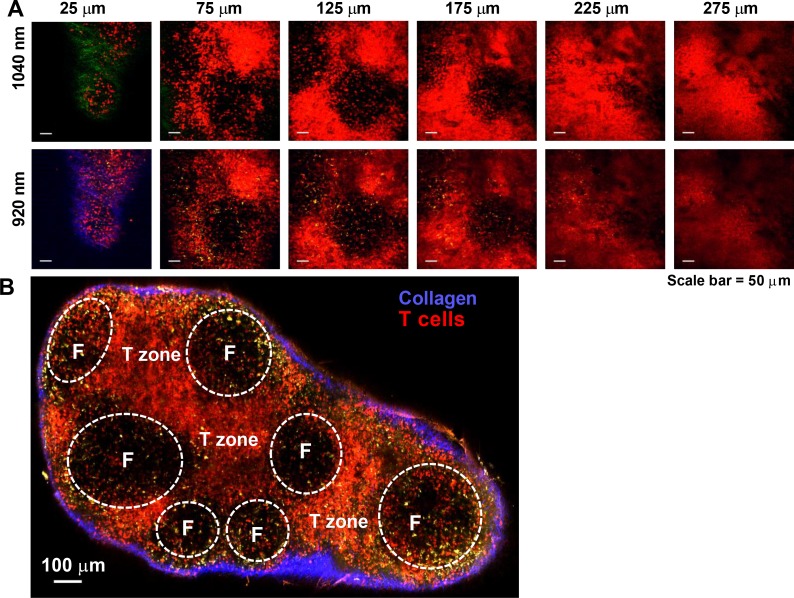
Imaging lymph nodes of Cd4-Salsa6f homozygous mice. Cre-mediated expression of Salsa6f in Cd4 T cells reveals endogenous T cell labeling in lymph node. (**A**) Two-photon images of explanted lymph node from Cd4-Salsa6f (Hom) mouse at various depths (indicated above each frame); 1040 nm femtosecond excitation (top row) or 920 nm excitation (bottom row). Second harmonic signal from collagen fibers is collected in green for 1040 nm excitation and in blue for 920 nm excitation. Salsa6f cells are readily detected up to 275 µm deep. (**B**) Montage image of a Cd4-Salsa6f (Hom) lymph node at 100 µm depth, imaged using 920 nm excitation showing Salsa6f^+^ cells in red, autofluorescent structures in yellow, and the capsular boundary shown in blue (second-harmonic signal). B cell follicles (F) are outlined. Scale bar = 100 µm.

**Figure 9—figure supplement 2.**
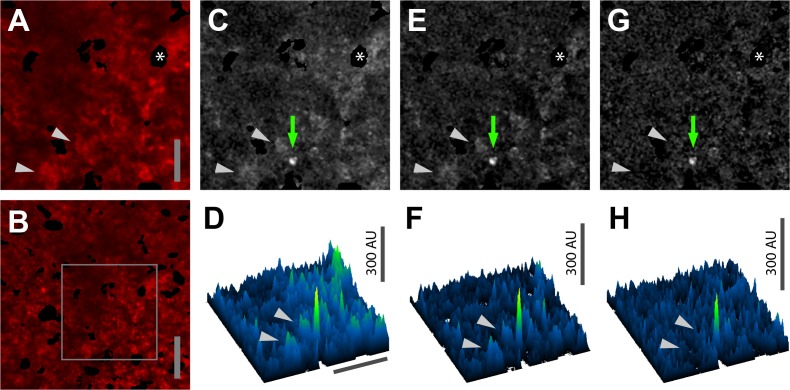
Subtraction of red channel fluorescence improves detection of Salsa6f Ca^2+^signals. (**A, B**) Median filtered, maximum intensity projection of a red channel image from a single time point of an explanted lymph node from a Cd4-Salsa6f (Hom) mouse. Panel **A** is enlarged and cropped from panel **B** (gray rectangle). (**C, E, G**) Green channel images corresponding to **A** with different image processing protocols. (**C**) Maximum intensity projection without further processing. (**E**) Maximum intensity projection after subtraction of the average of all green channel frames. (**G**) Maximum intensity projection after subtraction of the corresponding scaled red channel image and subtraction of the subsequent average from all green channel frames. Green arrows in **C,E,G** indicate a subcellular Ca^2+^ signal. (**D, F, H**) Surface plots corresponding to the images in **C,E,G**, respectively, showing the subcellular Ca^2+^ signal as a green peak. Gray arrowheads indicate nearby background cell fluorescence that is progressively removed by image processing. Asterisk indicates a region containing an autofluorescent cell that has been cropped out. Scale bar in **A** is 25 µm (applies to **C,E,G**); scale bar in **B** and the horizontal scale bar in **D** are 50 µm (applies to **F,H**).

**Video 6.** Lymph nodes from Cd4-Salsa6f (Hom) mice exhibit cell-wide and subcellular Ca^2+^signals. Time shown in hr:min:s; images were acquired at 5 s intervals. Play back speed = 50 frames per second. Red channel is turned off after beginning to facilitate visualization of green signals. Video corresponds to Figure 9B.

Because T cells move rapidly and are not uniformly distributed in lymph nodes, we developed an image processing approach in order to minimize fluctuations in background fluorescence and in order to sensitively identify cell-wide Ca^2+^ signals and sparkles (Figure 9—figure supplement 2). Based on the one-to-one correspondence of tdTomato and GCaMP6f, we estimated and subtracted out fluctuations in green fluorescence due to cell movement and distribution. After processing, sparkles were found to occur widely across the imaging field (Figure 9E–G), and many reached a characteristic maximum intensity (compare Figure 9F and G). The brightness of sparkles and the uniformity of background fluorescence allowed us to use a stringent threshold of signals exceeding 5.4 SD of the background noise level to systematically identify bright sparkles. Hundreds of events with a mean amplitude of 6.5 SD above background were observed in each 25 min imaging session (one image stack every five seconds), whereas fewer than one event exceeding our threshold was expected to occur from random noise fluctuations. Sparkles were more frequent than cell-wide transients (Figure 9H), extended over a median area of 1.9 µm^2^ (n = 441 sparkles from three lymph nodes; Figure 9I), and usually lasted for only one or two consecutive frames (Figure 9J). Sparkle trace shape differs from that expected for autofluorescent cell processes drifting into the imaging field (Figure 9K). Taken together, these observations suggest that sparkles correspond to local Ca^2+^ signals restricted to small subcellular domains of T cells migrating through the lymph node.

**Figure 10.**
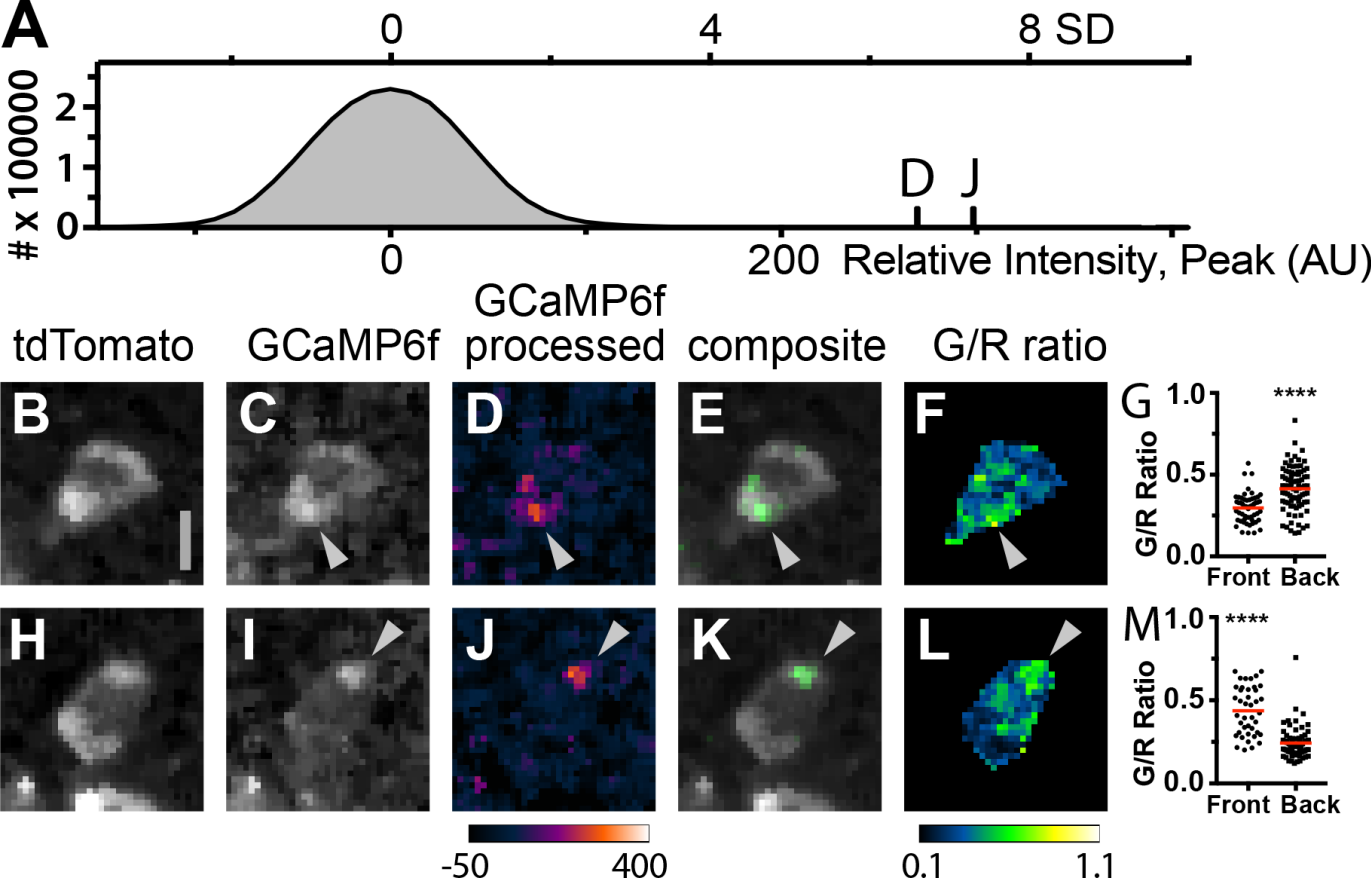
Subcellular Ca^2+^signals map to different regions of motile, adoptively transferred Cd4-Salsa6f (Hom) T cells. (**A**) Histogram of green channel pixel intensities from a representative region of a time-lapse image series of Cd4-Salsa6f (Hom) T cells in a wild-type lymph node after adoptive transfer. Vertical marks indicate the peak intensities of the fluorescence transients shown in **D** and **J**. (**B–F, H–L**) Two Cd4-Salsa6f (Hom) T cells imaged in a wild-type lymph node after adoptive transfer (same lymph node as in **A**). Red (**B,H**) and green (**C,I**) channel fluorescence images. (**D,J**) Corresponding pseudocolored green channel images processed as in Figure 9—figure supplement 2. (**E,K**) Corresponding composite image of gray pseudocolored red channel image with green channel processed image. (**F,L**) Ratiometric images of the green divided by the red channel fluorescence image. Gray arrowheads denote local Ca^2+^ signals at the back (**C–F**) and front (**I–L**) of motile T cells. Look-up table for **D** and **J**) corresponds to Arbitrary Units; look-up table for **F** and **L** corresponds to green-to-red ratio. Both cells are oriented with their front toward the top of the image. Scale bar in A is 5 µm (applies to B-H, **H–L**). (**G,M**) Scatter plots of G/R ratio for individual pixels in the front and back of Cd4-Salsa6f T cells shown in **B–F** and **H–L**, respectively. Red lines indicate median values. (****) indicates p<0.0001, Mann Whitney test.

The high density of expressing fluorescent T cells in the lymph nodes of Cd4-Salsa6f (Hom) mice makes it difficult to visualize and study subcellular Ca^2+^ signals within individual cells. We thus adoptively transferred Cd4-Salsa6f T cells into wild-type recipients so these cells could be viewed in isolation at low density. Small green fluorescence transients seen in lymph nodes after adoptive transfer were similar in intensity to transients seen in Cd4-Salsa6f (Hom) lymph nodes (Figure 10A). When small, bright, and brief green fluorescence signals were traced back, they were found to originate in red fluorescent Cd4-Salsa6f (Hom) T cells (Figure 10B–M). Cell movement was used to define the front and back of labeled T cells for mapping the subcellular location of Ca^2+^ signals. Some sparkles, identified in processed green channel images, were found to selectively localize to the front or back of motile T cells in which both the cell front and back were clearly seen (Figure 10D,E,J,K). Green-red channel ratiometric images, enabled by Salsa6f labeling, confirmed differences in Ca^2+^ concentration between front and back, although regions of elevated Ca^2+^ were also observed flanking the nucleus (Figure 10F,L). Differences in green-red channel ratiometric pixel intensities between front and back were highly significant (Figure 10G,M; p<0.0001, Mann–Whitney test). Thus, motile T cells exhibit Ca^2+^ signals that are largely restricted to subregions of the cytoplasm, and these Ca^2+^ signals – sparkles – were identified with high signal-to-noise ratio by Salsa6f expression. In a companion paper (Dong et al., 2017), we use Salsa6f transgenic mice to consider the relationship between Ca^2+^ signals, both cell-wide and local, and T-cell motility in the lymph node.

## Discussion

We introduce Salsa6f, a novel, ratiometric genetically-encoded Ca^2+^ probe. Salsa6f is a fusion of the high-performing green fluorescent GECI GCaMP6f and the bright red fluorescent tdTomato. This simple modification imparts powerful capabilities, which facilitate tracking cells in the absence of Ca^2+^ signaling, enable ratiometric imaging to eliminate motility artifacts, and permit convenient single-wavelength femtosecond excitation for two-photon microscopy. Salsa6f addresses a key weakness of single fluorescent protein-based GECIs by enabling tracking of motile cells and identification of cell morphology, even at basal Ca^2+^ levels when GCaMP6f fluorescence is very weak. We generated a transgenic reporter mouse with Cre-dependent expression of Salsa6f, enabling Ca^2+^ signals to be imaged in specific, genetically defined cell types. Transgenic expression of Salsa6f brings the power of ratiometric chemical Ca^2+^ indicators to imaging cellular Ca^2+^ signals amid the complex tissue environments found in vivo.

Salsa6f preserves the exceptional performance of GCaMP6f, which in the presence of high levels of Ca^2+^ is as bright as the standard high-performing green fluorescent protein, EGFP (Chen et al., 2013). We find that Salsa6f possesses a dynamic range similar to GCaMP6f, and both are superior to FRET-based GECIs (Heim et al., 2007; Thestrup et al., 2014). The Ca^2+^ affinity of Salsa6f, 160–300 nM, is well suited to detecting a variety of cellular Ca^2+^ signals. Inclusion of tdTomato in Salsa6f enables ratiometric imaging, calibration, and measurement of Ca^2+^ concentrations within cells. Moreover, Salsa6f is distributed uniformly throughout the cytosol; its exclusion from the nucleus provides reliable and selective reporting of cytosolic Ca^2+^ signaling. This is in contrast to the recently developed PC::G5-tdT mouse strain in which the tdTomato is found throughout the cell but the separately expressed GCaMP5G is excluded from the nucleus (Gee et al., 2014).

We created a transgenic mouse strain in which Salsa6f is expressed under genetic control using the Rosa26*^Cre^* recombinase system, and we used this system to label immune cells that express Cd4. Salsa6f labeling enables readout of cytosolic Ca^2+^ dynamics in T cells in vitro with high dynamic range, without the handling and potential toxicity associated with loading of chemical Ca^2+^ indicators. Indeed, a concern with any Ca^2+^ indicator is whether it may perturb cell function by buffering free Ca^2+^ ions or other means. In Cd4^+^ immune cells, we found no effects of Salsa6f expression, even in Cd4-Salsa6f (Hom) mice, with respect to cellular phenotype, cell proliferation, differentiation, and, in our companion paper (Dong et al., 2017), homing and T-cell motility.

Salsa6f was used to detect Ca^2+^ influx due to direct activation of SOCE, TCR stimulation, and an activator of Piezo1 channels – the latter observed in T cells for the first time to our knowledge. We also detected differences in patterns of Ca^2+^ signaling between naive T cells, Th17 cells, and iTregs. These experiments demonstrate the sensitivity, brightness, uniformity of labeling, and ease of detecting dynamic Ca^2+^ signals using Salsa6f.

A primary advance of this work is to take the in vitro capabilities of an excellent Ca^2+^ indicator and bring these into the realm of in vivo imaging. Within tissues, nearby cells exhibit differences, ranging from subtle to dramatic, in morphology, connectivity, and molecular profile. The red fluorescence of Salsa6f, combined with genetic Salsa6f labeling, associates these characteristics with readout of cellular Ca^2+^ signaling. Both the tdTomato and GCaMP6f in Salsa6f are excited well by a 920 nm femtosecond pulsed wavelength for two-photon imaging, enabling visualization hundreds of micrometers deep into lymph nodes. The immune system poses additional challenges for imaging because the constituent cells are highly motile. Indeed, direct cell-to-cell interactions of motile immune cells form the basis of immune surveillance (Mrass et al., 2010; Krummel et al., 2016). We were able to readily identify red fluorescent Salsa6f T cells easily in intact lymph nodes following adoptive transfer. Our images reveal uniform red fluorescence labeling by Salsa6f with clear subcellular morphology in imaging sessions encompassing hundreds of time lapse images. Moreover, Salsa6f offers the opportunity not only to record fluctuations in relative Ca^2+^ levels over time, but also to read out absolute Ca^2+^ concentrations within cells. We have measured the affinity of Salsa6f in intact cells; in principle, use of this approach will allow other microscope systems to be calibrated for measuring absolute Ca^2+^ concentrations with Salsa6f. Knowledge of absolute Ca^2+^ concentrations is necessary to develop quantitative models of Ca^2+^ signaling and cell behavior. Indeed, we demonstrate that clear Salsa6f ratio images can be generated from motile T cells in intact lymph nodes.

Our Salsa6f transgenic mouse line enables more sophisticated experimental approaches. One is the ability to detect rare Ca^2+^ signaling events. The high brightness and dynamic range of modern GECIs like Salsa6f contribute to detection of rare Ca^2+^ signaling events inside intact tissues or even whole transgenic animals (Kubo et al., 2014; Portugues et al., 2014). Ca^2+^ signals have been previously recorded within lymph nodes in adoptively transferred T cells transgenically expressing doxycycline-inducible mCameleon (Le Borgne et al., 2016), GCaMP3 and dsRed as separate proteins (Shulman et al., 2014), and virally transduced YC-nano-50^CD^ (Liu et al., 2015). Potential disadvantages of these probes include low dynamic range, nuclear expression of mCameleon and dsRed, the high Ca^2+^ affinity of YC-nano-50^CD^ (50 nM), and lack of ratiometric measurement. Imaging T cells transgenically expressing Salsa6f helps to overcome many of these limitations. Detecting rare events is made harder by inhomogeneities in cell populations of the lymph node as well as the movement of immune cells therein. Because of the one-to-one correspondence of tdTomato and GCaMP6f in Salsa6f, we were able to estimate and subtract resting GCaMP6f fluorescence even in motile cells. This approach substantially improves the uniformity of the fluorescence background upon which rare Ca^2+^ signaling events can be detected. Reliable and uniform cytosolic labeling contributes as well. Combined, these factors enabled us to detect not only sporadic cell-wide Ca^2+^ elevations, but also sparkles, much smaller sporadic local Ca^2+^ signals. The sensitivity and resolution of these images are sufficient to map local signals from intact lymph nodes to sub-regions of T cells. Moreover, while we focused upon the brightest local Ca^2+^ signals to demonstrate their existence, we expect that Salsa6f will enable even lower intensity Ca^2+^ signals to be linked to subcellular mechanisms and, ultimately, resulting cell behaviors. In a companion paper (Dong et al., 2017), we capitalize on the unique properties of Salsa6F to relate Ca^2+^ signals, both global and local, to T-cell motility in the intact lymph node.

## Materials and methods

**Key resources table:** 

Reagent type (species) or resource	Designation	Source or reference	Identifiers	Additional information
Recombinant DNA reagent	Salsa6f	This paper		Fusion of GCaMP6f to tdTomato via a V5 epitope linker (GCaMP6f-V5-tdTomato)
Recombinant DNA reagent	Gt(ROSA)26Sor5’-pCAG- FRT-LSL-Salsa6f-WPRE- bGHpA-AttB-FRT-NeoR- AttP-Gt(ROSA)26Sor3’ cassette	This paper		Salsa6f inserted into a Gt(ROSA)26Sor-pCAG- LSL-(Salsa6f)-WPRE- bGHpA-NeoR cassette.
Transgene (mouse)	*Gt(ROSA)26Sor^pCAG-FRT-LSL-^* *^Salsa6f-WPRE-bGHpA-AttB-FRT-NeoR-AttP^*	This paper		Allele with the above cassette targeted to the ROSA26 locus.
Transgene (mouse)	*Gt*(*ROSA*)*26Sor^pCAG-FRT-LSL-^* *^Salsa6f-WPRE-bGHpA-AttB/P^*	This paper		Same as above with Neomycin cassette deleted
Strain, strain background (mouse)	LSL-Salsa6f (F1), LSL-Sals6f (Hom)	This paper		Salsa6f transgene targeted to Rosa26 locus in JM8.N4 mouse embryonic stem (ES) cells. Positive chimeras bred to R26ΦC31o mice to produce LSL- Salsa6f F1 founders and homozygotic LSL- Salsa6f (Hom) mice. See Materials and methods for details.
Strain, strain background (mouse)	Cd4-Salsa6f (Het), Cd4-Salsa6f (Hom)	This paper		LSL-Salsa6f (Hom) mice crossed to *Cd4^Cre^* mice to produce heterozygotic and homozygotic *Salsa6f-* *Cd4^Cre^* mice (designated as Cd4- *Salsa6f^ ±^* and *Cd4-* *Salsa6f^+/+^* in the paper).
Strain, strain background (mouse)	*Cd4^Cre^ mice C57BL/6J*	Jackson #017336		
Strain, strain background (mouse)	C57BL/6J	Jackson #000664		
Cell line (human)	HEK293A	Invitrogen (#R705-07)		
Transfected construct (synthetic)	Salsa6f	This paper		see above for *Salsa6f* gene
Transfected construct (synthetic)	G-GECO1, B-GECO1, GCaMP6f, GCaMP6m, GCaMP6s	Addgene		
Antibody	anti-mouse IL-4, IL17A-APC (clone TC11-18H10.1), IFN-Pacific Blue (clone XMG1.2)	BioLegend		
Antibody	Foxp3-PE (clone FJK16s)	ThermoFisher Scientific		
Antibody	αCd3 and αCd28	Invivogen		
Antibody	αCd3 and αCd28 coated dynabeads	LifeTechnologies Corp.		
Peptide, recombinant protein	recombinant human TGFβ1	Tonbo Biosciences		
peptide, recombinant protein	recombinant mouse IL-12, IL-23, IL-1β, TGFβ	BioLegend		
Peptide, recombinant protein	recombinant human IL-2	BioLegend		
Commercial assay or kit	EasySep mouse naïve Cd4 T cell isolation kit	Stem Cell Technologies		
Commercial assay or kit	EasySep mouse Cd4 T cell isolation kit	Stem Cell Technologies		
Chemical compound, drug	Cell trace violet, eFluor 780	ThermoFisher Scientific		
Chemical compound, drug	Ionomycin, Thapsigargin, Retinoic Acid, PMA	Sigma Aldrich		
Chemical compound, drug	Ghost dye 780	Biolegend		
Chemical compound, drug	Fura2-AM, Fluo-4 AM	ThermoFisher Scientific		
Software, algorithm	ImageJ/Fiji	NIH		
Software, algorithm	IMARIS	Bitplane		
Other	35 mm glass chamber	LabTek, ThermoFisher Scientific		
Other	RPMI cell culture medium	Lonza		

### GECI screening and Salsa6f plasmid generation

Plasmids encoding GECIs (GECO and GCaMP6) were obtained from Addgene for screening in live cells. HEK 293A cells (Invitrogen- Life Technologies # R705-07) were screened for viruses and mycoplasma, split and frozen into working stocks, cultured using aseptic techniques, and used to evaluate candidate genetically encoded Ca^2+^ indicators. Each probe was cotransfected with Orai1 and STIM1 into HEK 293A cells using Lipofectamine 2000 (Invitrogen, Carlsbad, CA) for 48 hr before screening on an epifluorescence microscope. For construction of Salsa6f, a plasmid for tdTomato (Addgene, Cambridge, MA) and the pEGP-N1 vector (Clontech, Mountain View, CA) was used as a backbone. GCaMP6f was amplified via PCR with N- and C-terminal primers (5’ CACAACCGGTCGCCACCATGGTCGACTCATCACGTC 3’ and 5’ AGTCGCGGCCGCTTTAAAGCTTCGCTGTCATCATTTGTAC 3’) and ligated into pEGFP-N1 at the AgeI/NotI sites to replace the eGFP gene, while tdTomato was amplified via PCR with N- and C-terminal primers (5’ ATCCGCTAGCGCTACCGGTCGCC 3’ and 5’ TAACGAGATCTGCTTGTACAGCTCGTCCATGCC 3’) and ligated into the backbone at the NheI/BglII sites. An oligo containing the V5 epitope tag was synthesized with sense and antisense strands (5’ GATCTCGGGTAAGCCTATCCCTAACCCTCTCCTCGGTCTCGATTCTACG 3’ and 5’ GATCCGTAGAATCGAGACCGAGGAGAGGGTTAGGGATAGGCTTACCCGA 3’) and ligated into the backbone at the BglII/BamHI sites, linking tdTomato to GCaMP6f and creating Salsa6f. The amplified regions of the construct were verified by sequencing (Eton Bioscience Inc., San Diego, CA). This plasmid, driven by the CMV promoter, was used for transient transfections in HEK 293A cells with Lipofectamine 2000 and in primary human T cells with Amaxa Nucleofection.

### Transgenic mouse generation and breeding

The transgenic cassette in Figure 2B was generated by inserting Salsa6f, from the plasmid described above, into the Ai38 vector (Addgene Plasmid #34883) and replacing GCaMP3. The final targeting vector included the CAG (cytomegalovirus early enhancer/chicken β-actin) promoter, an LSL sequence with LoxP-STOP-LoxP, the Salsa6f probe (tdTomato-V5-GCaMP6f), the woodchuck hepatitis virus posttranscriptional regulatory element (WPRE), and a neomycin resistance gene (NeoR), all flanked by 5’ and 3’ Rosa26 homology arms of 1.1 and 4.3 kb. The targeting vector was linearized with PvuI and electroporated into JM8.N4 mouse embryonic stem (ES) cells of C57BL/6N background. Following selection with G418, clones carrying the *Gt(ROSA)26Sor^pCAG-FRT-LSL-Salsa6f-WPRE-bGHpA-AttB-FRT-NeoR-AttP^* allele were screened by Southern blotting after digestion with HindIII for the 5’ end or BglI for the 3’ end. Four correctly targeted clones were expanded and checked by chromosome counting, then two clones with >90% euploidy were further expanded and injected into C57BL/6J blastocysts for implantation into pseudopregnant foster mothers. Presence of the Salsa6f transgenic cassette was detected in the resulting chimeric pups by PCR screening for the *Nnt* gene, as the initial JM8.N4 ES cells are *Nnt*^+/+^ while the C57BL/6J blastocysts are *Nnt*^-/-^. Finally, positive chimeras were bred to R26ΦC31o mice (JAX #007743) to remove the neomycin resistance gene flanked by AttB and AttP sites in the original transgenic cassette, and to produce F1 founders carrying the allele *Gt(ROSA)26Sor^pCAG-FRT-LSL-Salsa6f-WPRE-bGHpA-AttB/P^* at the Rosa26 locus. These F1 founders were then bred to homozygosity to generate LSL-Salsa6f (Hom) mice, and subsequently crossed to homozygotic *Cd4^Cre^* mice (JAX #017336) to generate Cd4-Salsa6f (Het) mice expressing Salsa6f only in T cells. *Cd4-Salsa6f* mice were further bred to generate homozygotic Cd4-Salsa6f (Hom) mice for increased Salsa6f expression and fluorescence.

### T-cell proliferation and differentiation

*For T-cell proliferation*: Cd4 T cells were isolated from spleen and lymph nodes of 6–10 week old mice using negative selection (StemCell Technologies, Cambridge, MA). CellTrace Violet (CTV)-labeled T cells were co-cultured with αCd3/Cd28 coated dynabeads (Life Technologies Corp., Grand Island, NY) at 1:1 ratio according to the manufacturer’s protocol in a U bottom 96 well plate. *For T cell differentiation*: Naive Cd4 T cells were differentiated on activating polystyrene surface (Corning Inc., Corning, NY) with plate-bound αCd3 (2.5 µg/ml) and αCd28 (2.5 µg/ml) in the presence of cytokines for 6 days (Yosef et al., 2013). For Th1 differentiation: 25 ng/mL rmIL-12 (BioLegend, San Diego, CA), 10 µg/mL αmouse IL4 (Biolegend). For Th17 differentiation: 2.5 ng/mL rhTGF-β1 (Tonbo Biosciences, San Diego, CA), 50 ng/mL rmIL-6 (Tonbo Biosciences), 25 ng/ml rmIL-23 (BioLegend), and 25 ng/ml rmIL-β1 (BioLegend). For iTreg differentiation: 10 ng/mL rhTGF-β1, 100 units/mL of rmIL-2 (BioLegend), 5 µM Retinoic Acid (Sigma, St. Louis, MO).

### Flow cytometry

CTV dilution assay was performed in live cells (Fixable Viability Dye eFluor 780 negative gating; Thermofisher Scientific Inc., Grand Island, NY). To detect intracellular cytokines, 6 day differentiated cells were stimulated in with 25 ng of phorbol 12-myristate 13-acetate (PMA), 1 µg ionomycin (Sigma), and monensin (Golgistop BD biosciences) for 4 hr at 37°C. Dead cells were labeled with Ghost dye 780 (BioLegend), then washed, fixed, permeabilized using FoxP3 staining buffer set (Thermofisher Inc). The following antibodies were used to detect intracellular cytokines: IL-17A-APC (clone TC11-18H10.1, BioLegend); IFNγ-Pacific Blue (clone XMG1.2, BioLegend); Foxp3-PE (clone FJK16s, Thermofisher Scientific Inc.); in permeabilization buffer (eBioscience). Data were acquired using NovoCyte flow cytometer (ACEA Biosciences) and analyzed using FlowJo.

### T-cell preparation for live cell imaging

Cd4 T cells were activated by plating on six-well plates coated overnight with 2.5 μg/mL αCd3/αCd28 (Invivogen, San Diego, CA) at 4°C. Cells were cultured in RPMI medium (Lonza) containing 10% FCS, L-glutamine, Non-essential amino acids, Sodium pyruvate, β-mercaptoethanol and 50 U/mL of IL-2 at 37°C in 5% CO_2_ incubator. Following 2 days of culture, cells were plated on either poly-L-lysine or 1 μg/mL α-Cd3/α28 coated 35-mm glass chambers (Lab-Tek, Thermofisher Inc.) for imaging. RPMI medium with 2% FCS and L-glutamine containing 2 mM Ca^2+^ was used for imaging experiments. For experiments involving calibration and characterization of the Salsa6f probe in Cd4-Salsa6f cells, Ringer solution containing various concentrations of Ca^2+^ was used. For Ca^2+^ imaging of different T-cell subsets, Th17 cells and iTregs were differentiated as described above.

### Confocal imaging and analysis

For Ca^2+^ imaging of Cd4^+^ T cells from Cd4-Salsa6f mice, we used an Olympus Fluoview FV3000RS confocal laser scanning microscope, equipped with high-speed resonance scanner and the IX3-ZDC2 Z-drift compensator (Olympus Corp., Waltham, MA). Diode lasers (488 and 561 nm) were used for excitation, and two high-sensitivity cooled GaAsP PMTs were used for detection. Cells were imaged using the Olympus 40x silicone oil objective (NA 1.25), by taking five slice z-stacks at 2 µm/step, at 5 s intervals, for up to 20 min. Temperature, humidity, and CO_2_ were maintained using a Tokai-Hit WSKM-F1 stagetop incubator. Data were processed and analyzed using Imaris and ImageJ software. Calcium imaging experiments were done at 37°C on 2-day-activated Cd4^+^ T cells from Cd4-Salsa6f (Het) mice, unless otherwise indicated. Salsa6f calibration experiments were done at room temperature.

### Two-photon microscopy

Lymph nodes images were acquired using a custom-built two photon microscope based on Olympus BX51 upright frame, Motorized ZDeck stage (Prior, Rockland, MA), with excitation generated by a tunable Chameleon femtosecond laser (Coherent, Santa Clara, CA) (Miller et al., 2002). The following wavelengths were used to excite single or combination of fluorophores: 920 nm to excite tdTomato and GCaMP6f; 1040 nm to excite tdTomato alone. 495 nm and 538 nm dichroic filters were arranged in series to separate blue, green and red signals. Two-photon excitation maxima of tdTomato and GCaMP6f are 1040 and 920 nm, respectively (Drobizhev et al., 2011; Chen et al., 2013). Using 1040 nm excitation, tdTomato signals were readily detected up to 300 µm depth; however, 1040 is not ideal to image Salsa6f because: 1) Collagen fibers generate second harmonic at 520 nm when excited with 1040 nm, which interferes with simultaneous detection of GCaMP6f (emission maxima, 509 nm) and 2) 1040 nm does not excite GCaMP6f (Figure 9—figure supplement 1A, top row). Alternatively, 920 nm optimally excites GCaMP6f, and excites tdTomato sufficiently, and Salsa6f signals were detected up to 300 µm depth, while second harmonic collagen signals (460 nm) can be easily separated into blue channel (Figure 9—figure supplement 1A, bottom row). Additionally, autofluorescent structures (LN resident DCs and fibroblastic reticular cells) show up as yellow bodies when excited with 920 nm, which serve as a guide to locate the T cell zone (Figure 9—figure supplement 1B). Therefore, 920 nm is the ideal two-photon excitation wavelength for simultaneous imaging of tdTomato and GCaMP6f as component parts of Salsa6f.

Lymph nodes were oriented with the hilum away from the water dipping microscope objective (Nikon 25x, NA 1.05). The node was maintained at 36–37°C by perfusion with medium (RPMI) bubbled with medical grade carbogen (95% O_2_ and 5% CO_2_) using a peristaltic heated perfusion system (Warner Instruments), with thermocouple-based temperature sensors placed next to the tissue in a custom built chamber. 3D image stacks of x = 250 μm, y = 250 μm, and z = 20 or 52 μm (4 μm step size) were sequentially acquired at 5 or 11 s intervals, respectively, using image acquisition software Slidebook (Intelligent Imaging Innovations) as described previously (Matheu et al., 2015). This volume collection was repeated for up to 40 min to create a 4D data set.

### Data analysis and statistical testing

Graphpad Prism was used for statistical analysis and generating figures. p values are indicated in figures: ns p>0.05, *p<0.05; **p<0.01; ***p<0.001; and ****p<0.0001.

### Detection of Ca^2+^ signals in lymph nodes

Stacks of six optical sections 4 µm apart from the T-zone of Cd4-Salsa6f (Hom) lymph nodes were acquired once every 5 s at a resolution of 0.488 or 0.684 µm per pixel. Maximum intensity projections of 1 pixel radius median-filtered images were used for subsequent processing and analysis. Autofluorescent cells were identified by averaging the red or green time lapse image stacks and automated local thresholding (Bernsen five pixel radius) using the public domain image processing program ImageJ. Autofluorescent cell masks were dilated by four pixels, regions exhibiting less contrast and detail due to light scattering manually masked to produce the final time lapse image mask. Red (tdTomato) channel fluorescence from Salsa6f corresponding to green (GCaMP6f) channel resting state fluorescence was determined to be fivefold higher using our standard two-photon microscope acquisition settings. Final green images were produced by subtracting a 0.2x scaled red channel image, and subsequently subtracting out the average of all green channel time lapse images. The standard deviation (SD) of each masked green channel time lapse image stack was used to determine thresholds for local (sparkle) and cell-wide Ca^2+^ events. Thresholds for detection of local and cell-wide Ca^2+^ events were 5.4 and 2.1 SD and 1.4 µm^2^ and 25 µm^2^, respectively. Local Ca^2+^ events were excluded from the set of sparkles if they coincided with a cell-wide event. Frequency of background events was calculated using a standard normal distribution with a Z-score corresponding to the average intensity of local events (6.5 SD), which was 1 in 2 × 10^10^ pixels (WolframAlpha). The front and back of motile T cells were traced manually from red channel TdTomato images. Individual pixel intensities from the front and back regions of interest were plotted and compared using the non-parametric Mann–Whitney test in Graphpad Prism.

## Additional files

Transparent reporting form
